# Shaping sustainable paths for HIV/AIDS funding: a review and reminder

**DOI:** 10.1097/MS9.0000000000002976

**Published:** 2025-02-27

**Authors:** Chukwuka Elendu, Dependable C. Amaechi, Tochi C. Elendu, Emmanuel C. Amaechi, Ijeoma D. Elendu, Kenneth N. Akpa, Praise O. Oloyede, Michael O. Adegbola, Omoyelemi F. Idowu

**Affiliations:** a Department of Internal Medicine, Federal University Teaching Hospital, Owerri, Nigeria; b Department of Medicine, Igbinedion University, Okada, Nigeria; c Department of Nursing Science, Imo State University, Owerri, Nigeria; d Department of Medicine, Madonna University, Elele, Nigeria; e Department of Internal Medicine, University of Port Harcourt Teaching Hospital, Choba, Nigeria; f Department of Internal Medicine, Babcock University Teaching Hospital, Ilishan-Remo, Nigeria; g Department of Medicine, Olabisi Onabanjo University, Ago-Iwoye, Nigeria

**Keywords:** global health financing, HIV/AIDS funding, innovative funding models, public-private partnerships, sustainable financial strategies

## Abstract

The fight against human immunodeficiency virus/acquired immunodeficiency syndrome (HIV/AIDS) has made significant progress over the past decades, yet sustainable funding remains a critical challenge. Despite advances in medical treatments and prevention methods, the financial resources needed to combat the epidemic consistently face uncertainties and shortfalls. As of 2023, approximately 37.7 million people are living with HIV/AIDS globally, with 1.5 million new infections reported annually. Sub-Saharan Africa remains the hardest-hit region, accounting for 67% of the global HIV burden. This paper examines the current state of HIV/AIDS funding, identifying key gaps and challenges in maintaining adequate financial resources. It highlights the effects of funding fluctuations on treatment accessibility, prevention programs, and research, stressing the urgent need for diversified and innovative financing mechanisms. The paper offers actionable insights into sustainable funding strategies by analyzing successful models such as public-private partnerships and social impact bonds.

This review aims to inform policymakers, stakeholders, and the global community about the financial barriers to HIV/AIDS management and advocate for coordinated efforts to secure stable funding pathways. Ensuring consistent financial support is vital to preserving hard-won progress, expanding access to care, and achieving the ultimate goal of an AIDS-free generation.

## Introduction and background

Human immunodeficiency virus (HIV) and acquired immunodeficiency syndrome (AIDS) constitute one of the most significant global health challenges of modern times. Since its identification in the early 1980s, HIV/AIDS has profoundly impacted communities worldwide, particularly in sub-Saharan Africa, where the epidemic has been most severe^[1]^. As of 2023, approximately 37.7 million people are living with HIV/AIDS globally, with 1.5 million new infections reported annually^[2]^. This paper examines the various funding mechanisms for HIV/AIDS, emphasizing the necessity for sustainable financial strategies to address the ongoing challenges of the epidemic. The discovery of HIV/AIDS in the early 1980s marked the beginning of a global health crisis that rapidly spread across continents. Initial cases were reported among gay men in the United States, leading to widespread fear and stigma^[3]^. By 1984, scientists had identified HIV as the causative agent of AIDS, a breakthrough that spurred research into the virus’s transmission, pathogenesis, and potential treatments^[4]^. Despite early advancements, the epidemic continued to grow, with sub-Saharan Africa becoming the most affected region. The spread of HIV in this region was facilitated by socio-economic factors, including poverty, lack of education, gender inequality, and limited access to healthcare services^[5,6]^. HIV is a retrovirus that targets the immune system, specifically CD4+ T cells, leading to their gradual depletion and the eventual collapse of the immune response if untreated. This immunodeficiency makes individuals susceptible to opportunistic infections and certain cancers, which are the primary causes of morbidity and mortality in AIDS patients^[7]^. The understanding of HIV’s pathogenesis has been crucial in developing antiretroviral therapies (ART) that suppress viral replication and restore immune function. The introduction of ART in the mid-1990s transformed HIV from a fatal disease into a manageable chronic condition, significantly reducing AIDS-related deaths^[8]^. Despite these advancements, the global distribution of HIV/AIDS remains uneven, with sub-Saharan Africa bearing the highest burden. This region accounts for approximately 67% of all people living with HIV and over half of all new infections^[9]^. The epidemic in sub-Saharan Africa is characterized by high prevalence rates among women and young people, driven by factors such as gender-based violence, economic dependence on men, and limited access to reproductive health services^[10,11]^. Efforts to address the epidemic in this region have focused on expanding ART coverage, promoting gender equality, and implementing prevention programs, such as voluntary medical male circumcision and pre-exposure prophylaxis (PrEP)^[12,13]^.

**HIGHLIGHTS**

- Sustained funding is crucial for the fight against HIV/AIDS, yet financial support faces growing challenges.
- Reduced funding could impact access to treatment, prevention, and research efforts.
- Innovative financing strategies are needed to secure sustainable HIV/AIDS programs.

In contrast, the HIV/AIDS epidemic in Eastern Europe and Central Asia has been primarily driven by injecting drug use. Harm reduction strategies, including needle exchange programs and opioid substitution therapy, have proven effective in reducing HIV transmission among people who inject drugs (PWID)^[14,15]^. However, political instability, stigma, and punitive drug policies have hindered the implementation of these interventions in many countries, leading to continued high rates of HIV transmission in this region^[16]^. Asia and the Pacific region present a diverse epidemiological landscape, with concentrated epidemics among key populations such as men who have sex with men (MSM), sex workers, and transgender individuals. Cultural and legal barriers, along with stigma and discrimination, pose significant challenges to HIV prevention and treatment efforts in this region^[17,18]^. Innovative community-based interventions and targeted outreach programs have shown promise in addressing these barriers and improving access to HIV services for marginalized groups^[19]^. Western and Central Europe and North America have experienced significant declines in HIV incidence and AIDS-related deaths thanks to widespread access to ART and comprehensive prevention strategies, including PrEP and harm reduction services^[20]^. However, disparities persist, particularly among racial and ethnic minorities, migrants, and MSM. These disparities highlight the need for ongoing efforts to address social determinants of health and ensure equitable access to HIV services^[21,22]^. Latin America and the Caribbean region face a mixed HIV epidemic, with high prevalence rates in some countries and low rates in others. Key populations, including MSM, sex workers, and transgender women, are disproportionately affected^[23]^. Stigma, discrimination, and socio-economic inequalities continue to hinder HIV prevention and treatment efforts in this region. Community-led initiatives and regional collaborations have been instrumental in promoting human rights and improving access to HIV services^[24,25]^. The Middle East and North Africa (MENA) region has a relatively low HIV prevalence, but the epidemic is concentrated among key populations such as MSM, PWID, and sex workers. Stigma, criminalization, and limited access to healthcare services are significant barriers to effective HIV response in this region^[26]^. Advocacy for human rights and the implementation of harm reduction and targeted prevention programs are critical to addressing the HIV epidemic in MENA^[27]^.

The global response to HIV/AIDS has been shaped by significant milestones and initiatives, including the establishment of the Joint United Nations Programme on HIV/AIDS (UNAIDS) in 1996 and the launch of the President’s Emergency Plan for AIDS Relief (PEPFAR) in 2003^[28,29]^. These initiatives have mobilized substantial financial and technical resources to support HIV prevention, treatment, and care programs worldwide. The Global Fund to Fight AIDS, Tuberculosis, and Malaria, established in 2002, has also played a pivotal role in financing HIV programs in low- and middle-income countries^[30]^. Sustainable funding is essential to maintain and expand the progress achieved in the global fight against HIV/AIDS. Funding supports critical activities, including the procurement of ART, implementation of prevention programs, and strengthening of healthcare systems^[31]^. However, funding gaps and donor fatigue pose significant challenges to sustaining the HIV response. The COVID-19 pandemic has further strained resources and disrupted HIV services, underscoring the need for resilient and adaptable funding mechanisms^[32,33]^. Innovative financing approaches, such as public-private partnerships (PPPs), social impact bonds, and domestic resource mobilization, are being explored to ensure sustainable funding for HIV/AIDS programs^[34]^. These approaches aim to leverage diverse funding sources, enhance efficiency, and promote accountability in the use of resources.

Additionally, integrating HIV services with other health and social services can improve cost-effectiveness and address the broader health needs of people living with HIV^[35,36]^. The role of research and development in advancing the HIV response cannot be overstated. Continued investment in biomedical research is crucial for developing new prevention technologies, such as vaccines and long-acting injectable ART, and improving existing treatment regimens^[37]^. Operational research is also essential to optimize the delivery of HIV services and address implementation challenges in diverse settings^[38]^. Community engagement and leadership are vital components of an effective HIV response. People living with HIV, key populations, and affected communities play a crucial role in designing, implementing, and monitoring HIV programs^[39]^. Their involvement ensures that interventions are responsive to the needs and realities of those most affected by the epidemic and promotes greater ownership and sustainability of HIV services^[40]^. Human rights-based approaches are fundamental to addressing the structural drivers of the HIV epidemic and ensuring equitable access to HIV services. Legal and policy reforms, advocacy for non-discrimination, and efforts to reduce stigma are essential to creating an enabling environment for effective HIV prevention, treatment, and care^[41]^. Protecting the rights of key populations, including MSM, PWID, sex workers, and transgender individuals, is particularly critical to reducing their vulnerability to HIV and improving their health outcomes^[42]^. Education and awareness campaigns are essential tools for HIV prevention and reducing stigma. Comprehensive sexuality education, tailored to the needs of different age groups and cultural contexts, can empower individuals with knowledge and skills to protect themselves from HIV and promote healthy behaviors^[43]^. Media campaigns, peer education, and community outreach are effective strategies for raising awareness and promoting HIV testing and treatment^[44]^. The integration of HIV services into broader health systems is essential for improving the efficiency and sustainability of the HIV response.

Integrated service delivery models, such as one-stop clinics and community health centers, can enhance access to a range of health services, reduce stigma, and improve health outcomes for people living with HIV^[45]^. Strengthening health systems, including improving health infrastructure, training healthcare workers, and enhancing supply chain management, is critical to ensuring the effective delivery of HIV services^[46]^. Monitoring and evaluation (M&E) are key components of effective HIV programs. Robust M&E systems provide essential data for tracking progress, identifying gaps, and informing programmatic decisions^[47]^. Advances in digital health technologies, such as electronic health records and mobile health applications, offer new opportunities for improving M&E and enhancing the delivery of HIV services^[48]^. Collaboration and partnerships are vital for a coordinated and effective HIV response. Governments, international organizations, civil society, the private sector, and affected communities must work together to leverage their strengths and resources^[49]^. Global initiatives, such as the sustainable development goals (SDGs) and the 90-90-90 targets set by UNAIDS, provide a framework for aligning efforts and measuring progress towards ending the HIV epidemic^[50]^.

### Statement of concrete aim

This study aims to explore innovative strategies and pivotal interventions in global HIV/AIDS funding, focusing on enhancing sustainability and effectiveness. By dissecting fundamental funding mechanisms and examining successful models, this research seeks to uncover actionable insights that can propel global efforts toward achieving equitable access to prevention, treatment, and care services. Through a nuanced analysis of funding challenges and opportunities, this study aims to inspire transformative approaches that mitigate disparities and accelerate progress toward ending the HIV/AIDS epidemic by 2030.

### Data sources and collection

This review article utilized a comprehensive literature search to gather relevant data on the geographical distribution, epidemiology, and funding mechanisms for HIV/AIDS. Data sources included peer-reviewed journal articles, reports from international health organizations such as the World Health Organization (WHO) and the UNAIDS, and publications from governmental and non-governmental organizations. Statistical data were obtained from the latest UNAIDS Global AIDS Update and other reputable databases.

### Literature search strategy

The literature search used electronic databases such as PubMed, Scopus, and Google Scholar. Keywords used in the search included “HIV/AIDS epidemiology,” “HIV prevalence,” “HIV funding,” “antiretroviral therapy coverage,” “HIV key populations,” and “HIV interventions.” Articles from 2000 to 2023 were included to ensure the review covered the most recent data and trends. The search was also extended to include relevant grey literature, such as conference abstracts, policy briefs, and reports from international organizations.

#### Inclusion criteria

- Studies and reports that provided quantitative data on HIV/aids prevalence, incidence, and key populations.
- Publications that discussed funding mechanisms and interventions for HIV/AIDS.
- Articles and reports focused on specific regions or provided comparative analysis across different areas.

#### Exclusion criteria:

- Studies that did not provide primary data or were not relevant to the geographical distribution of HIV/AIDS.
- Articles published before 2000 unless they provided critical historical context.

### Data extraction and synthesis

Two researchers performed data extraction independently to minimize bias and ensure accuracy. The extracted data included prevalence, new infection rates, ART coverage, key affected populations, and significant regional challenges and interventions. The extracted data were then synthesized into a comprehensive table (Table 1) to facilitate comparative analysis and highlight regional disparities and trends.

**Table 1:** Geographical distribution of HIV/AIDS, including various aspects such as prevalence, incidence, key populations, and regional challenges

Region	Total population (millions)	HIV prevalence (%)	New infections (per year)	Key affected populations	ART coverage (%)	Major challenges	Notable interventions
Sub-Saharan Africa	1136	6.7	1 200 000	Women, Adolescents, MSM, PWID	76	Gender inequality, stigma, weak health systems	PEPFAR, Global Fund, PMTCT programs
Eastern Europe & Central Asia	245	0.9	190 000	PWID, MSM, prisoners	45	Political instability, limited harm reduction	Harm reduction programs, Global Fund
Asia & Pacific	4300	0.2	280 000	MSM, PWID, sex workers, migrants	54	Stigma, migration, diverse epidemic trends	Harm reduction, community-based programs
Western & Central Europe & North America	1121	0.4	69 000	MSM, migrants, PWID	78	Stigma, health disparities, aging Population	PrEP, ART scale-up, health equity initiatives
Latin America	654	0.4	100 000	MSM, sex workers, transgender women	61	Stigma, socioeconomic inequality	Community health programs, PrEP
Middle East & North Africa	370	0.1	19 000	MSM, PWID, sex workers, migrants	35	Stigma, criminalization, limited health services	Global Fund, advocacy for human rights
Caribbean	44	1.1	14 000	MSM, sex workers	63	Stigma, small population size, resource constraints	PEPFAR, regional collaboration
South America	428	0.5	120 000	MSM, PWID, sex workers	66	Stigma, economic disparities	National HIV/AIDS programs, PrEP

Sub-Saharan Africa remains the epicenter of the HIV/AIDS epidemic, with a staggering prevalence rate of 6.7% among a total population of approximately 1.136 billion people. This region accounts for the highest number of people living with HIV, as well as new infections, which stand at around 1.2 million annually. The epidemic in this region is primarily driven by heterosexual transmission, with women and adolescents being disproportionately affected. Additionally, men who have sex with men (MSM) and people who inject drugs (PWID) are significant key populations. Despite notable progress, including a 76% antiretroviral therapy (ART) coverage rate, challenges such as gender inequality, stigma, and weak health systems persist. Major regional interventions include PEPFAR, the Global Fund, and comprehensive prevention of mother-to-child transmission (PMTCT) programs, which have been instrumental in reducing new infections and improving treatment access. In Eastern Europe and Central Asia, the HIV prevalence rate stands at 0.9% among a population of 245 million, with approximately 190 000 new infections occurring annually. Key populations in this region include PWID, MSM, and prisoners. Political instability, limited harm reduction services, and high levels of stigma are significant barriers to effective HIV response. ART coverage in this region is relatively low at 45%, highlighting the need for expanded access to treatment. Harm reduction programs and support from the Global Fund are crucial interventions to address this region’s epidemic. The Asia and Pacific region, with a population of 4.3 billion, has a relatively low HIV prevalence rate of 0.2%, yet it still sees around 280 000 new infections each year. The epidemic here is diverse, affecting key populations such as MSM, PWID, sex workers, and migrants. The region faces significant challenges, including high levels of stigma, migration-related vulnerabilities, and varying epidemic trends across different countries. ART coverage is at 54%, indicating moderate success in treatment access. Still, there remains a need for greater efforts in harm reduction and community-based programs to reach the most vulnerable populations. Western and Central Europe and North America have a combined population of 1.121 billion and a prevalence rate of 0.4%, with 69 000 new infections annually. In these high-income regions, MSM, migrants, and PWID are the key affected populations. Despite relatively high ART coverage at 78%, challenges such as stigma, health disparities, and an aging population living with HIV persist. Interventions like pre-exposure prophylaxis (PrEP), ART scale-up, and health equity initiatives are vital in addressing the epidemic in these regions. Latin America, with a population of 654 million, has a prevalence rate of 0.4% and sees about 100 000 new infections each year. The epidemic is concentrated among key populations, including MSM, sex workers, and transgender women. Stigma and socioeconomic inequality are major challenges that hinder effective HIV response. ART coverage in the region is 61%, and interventions include community health programs and PrEP to reduce new infections and improve treatment outcomes. In the Middle East and North Africa, the prevalence rate is low at 0.1% among a population of 370 million, with approximately 19 000 new infections annually. Key populations such as MSM, PWID, sex workers, and migrants are most affected. The region faces significant challenges, including high levels of stigma, criminalization of key populations, and limited access to healthcare services. ART coverage is low at 35%, underscoring the need for expanded treatment and advocacy for human rights. Support from the Global Fund and other international initiatives is critical in addressing the epidemic in this region. The Caribbean, with a population of 44 million, has a prevalence rate of 1.1%, one of the highest outside sub-Saharan Africa, and sees about 14 000 new infections yearly. MSM and sex workers are key affected populations. Challenges in the region include stigma, small population size, and resource constraints. ART coverage is at 63%, and major interventions include PEPFAR and regional collaboration efforts to enhance HIV prevention and treatment services. South America, with a population of 428 million, has a prevalence rate of 0.5% and sees around 120 000 new infections annually. The epidemic primarily affects MSM, PWID, and sex workers. Stigma and economic disparities are significant challenges in addressing HIV in this region. ART coverage is at 66%, and interventions include national HIV/AIDS programs and PrEP to improve access to prevention and treatment services. Across all these regions, the burden of HIV/AIDS is shaped by a combination of social, economic, and structural factors that influence transmission dynamics and access to healthcare services. While significant progress has been made in expanding ART coverage and implementing effective prevention strategies, persistent challenges remain in achieving equitable access and addressing the unique needs of key populations. Efforts to combat HIV/AIDS globally must continue to focus on reducing stigma and discrimination, enhancing healthcare infrastructure, and ensuring that key populations have access to comprehensive prevention and treatment services. Additionally, sustained political commitment and financial support are crucial for maintaining and scaling up effective interventions. The global response to HIV/AIDS requires a coordinated and multisectoral approach that leverages the strengths of governments, civil society, international organizations, and communities affected by the epidemic. By addressing the underlying social determinants of health and promoting human rights, the international community can make significant strides towards ending the HIV/AIDS epidemic and improving the lives of millions of people worldwide. [Authors’ creations]

### Statistical analysis

Descriptive statistics summarized the prevalence, incidence, and ART coverage data across different regions. Comparative analysis was conducted to identify trends and disparities in HIV/AIDS epidemiology and funding. The study also examined the relationship between key populations and regional challenges and the impact of major interventions on epidemic control.

## Limitations

This review analyses HIV/AIDS funding mechanisms and their geographical distribution; however, several limitations must be acknowledged. One significant concern is the potential impact of publication bias. Reliance on published literature and reports may skew findings toward regions, programs, or funding models more likely to receive attention due to their perceived successes or innovations. As a result, critical insights from underreported areas or less successful interventions may be underrepresented, limiting the conclusions’ generalizability. Addressing this issue in future research requires the inclusion of gray literature, such as organizational reports, conference proceedings, and unpublished datasets, to provide a more balanced perspective. Regional data variability further complicates the analysis. Differences in data quality, collection methodologies, and reporting standards across various regions may affect the comparability of findings. This variability is particularly pronounced in low-income or high-burden areas where data infrastructure may need to be improved. Such disparities can lead to an uneven representation of regional funding needs and outcomes, potentially influencing policy recommendations that do not adequately address global inequities. Future research should advocate for establishing standardized data collection frameworks and reporting protocols to address this, fostering greater consistency and reliability in data across regions.

Moreover, the reliance on cross-sectional and secondary data limits the ability to assess funding sustainability over time. Longitudinal studies incorporating primary data collection could provide more detailed insights into funding mechanisms’ evolution and long-term effectiveness. Such approaches would also allow researchers to identify emerging trends and adapt funding strategies to changing epidemiological and economic conditions. By addressing these limitations, future research can offer a more nuanced understanding of HIV/AIDS funding and better support efforts to develop sustainable and equitable solutions.

### The global HIV/aids landscape

HIV and AIDS represent one of the most formidable challenges in global public health history, profoundly impacting societies worldwide since its emergence in the early 1980s^[1]^. The discovery of HIV as the causative agent of AIDS marked a pivotal moment in medical science, spearheaded by the pioneering research of Dr. Robert Gallo in the United States and Dr. Luc Montagnier in France^[2,3]^. Their groundbreaking work isolating and characterizing the virus laid the Foundation for subsequent efforts to understand its transmission, pathogenesis, and treatment. The initial cases of AIDS were predominantly identified among gay men in urban centers of the United States, presenting with rare opportunistic infections and malignancies that signaled severe immune suppression^[4]^. The mysterious and rapidly fatal nature of the disease sparked widespread fear and stigmatization, exacerbating social tensions and hindering public health responses^[5]^. By the mid-1980s, as the scale of the epidemic became apparent, AIDS had spread to diverse populations globally through various transmission routes, including blood transfusions, intravenous drug use, and heterosexual contact^[6]^. The global response to HIV/AIDS evolved amidst a backdrop of scientific uncertainty and socio-political challenges. Early efforts to combat the epidemic were hampered by limited knowledge of HIV transmission and the absence of effective treatments. The lack of a coordinated international response initially delayed decisive action, allowing the virus to proliferate unchecked in many regions^[7]^. In the absence of effective antiretroviral therapies (ART), prevention strategies focused primarily on behavioral interventions and promoting safe sex practices^[8]^. The landscape of HIV/AIDS funding began to shift significantly with the advent of antiretroviral therapy (ART) in the mid-1990s. The introduction of drugs such as zidovudine (AZT) and later protease inhibitors revolutionized HIV/AIDS management, transforming the disease from a death sentence to a chronic, manageable condition for those with access to treatment^[9]^. The success of ART in prolonging life expectancy and reducing AIDS-related mortality underscored the importance of scaling up treatment programs globally.

International efforts to address HIV/AIDS gained momentum with the establishment of the UNAIDS in 1996. UNAIDS was pivotal in coordinating global responses, advocating for increased funding, and setting ambitious targets for prevention, treatment, and care^[10]^. The 2001 United Nations General Assembly Special Session on HIV/AIDS (UNGASS) marked a watershed moment in galvanizing political commitment and mobilizing resources to combat the epidemic^[11]^. Throughout the 2000s and into the 2010s, the HIV/AIDS response witnessed unprecedented strides in scaling up access to treatment and prevention services, particularly in low- and middle-income countries heavily impacted by the epidemic. The launch of the PEPFAR in 2003 by the United States government and the establishment of the Global Fund to Fight AIDS, Tuberculosis, and Malaria in 2002 represented landmark initiatives in mobilizing substantial financial resources and technical assistance^[12,13]^. Despite these advancements, challenges persist in ensuring equitable access to HIV/AIDS services, particularly among marginalized populations and in resource-constrained settings. Economic disparities, political instability, and social stigma impede progress, exacerbating health inequities and hindering efforts to achieve global targets such as those outlined in the SDGs^[14]^. The sustainability of HIV/AIDS funding remains a critical concern, with donor fatigue and competing health priorities threatening to undermine gains made in the fight against the epidemic^[15]^. Looking forward, addressing the structural determinants of health, strengthening health systems, and promoting human rights are essential for sustaining progress towards ending the HIV/AIDS epidemic by 2030. The next phase of the global HIV/AIDS response must prioritize innovation, resilience, and collaboration across sectors to ensure that no one is left behind in the quest for a world free from HIV/AIDS.

Building upon the historical evolution of HIV/AIDS, understanding the current global burden of the epidemic is essential to contextualize ongoing challenges and responses. HIV and AIDS remain significant public health challenges worldwide, with profound implications for health outcomes, socio-economic development, and human rights. Since the early 1980s, the epidemic has dynamically evolved, impacting diverse populations and regions to varying extents^[1]^. Epidemiological trends continue to highlight the intricate interplay of biological, behavioral, social, and structural factors that shape the transmission and distribution of HIV/AIDS across different populations. Table 1 illustrates the diverse geographical distribution of HIV/AIDS, highlighting variations in prevalence, new infections, and key affected populations across different regions. It underscores each region’s challenges, from high prevalence rates in sub-Saharan Africa to concentrated epidemics among key populations in regions like Eastern Europe and Central Asia. The table also showcases the different ART coverage levels and the notable interventions implemented globally to combat the epidemic. As of 2023, an estimated 37.7 million people worldwide are living with HIV/AIDS, with sub-Saharan Africa bearing the greatest burden, accounting for approximately 67% of all people living with HIV globally^[2]^. Despite substantial progress in expanding access to ART and implementing effective prevention measures, the epidemic continues to disproportionately affect vulnerable and marginalized populations, including women, children, adolescents, MSM, transgender individuals, PWID, and sex workers^[3,4]^. Global epidemiological data reveal significant regional variations in HIV prevalence and incidence rates. Sub-Saharan Africa remains the most heavily affected region, with Southern and Eastern Africa experiencing the highest prevalence rates. Countries such as South Africa, Nigeria, Uganda, and Kenya have prevalence rates exceeding 10% among adults aged 15-49 years^[5]^. In contrast, other regions such as North America, Western Europe, and parts of Asia have lower prevalence rates but face localized epidemics among key populations^[6]^. Diverse patterns of transmission characterize the epidemiology of HIV/AIDS. Sexual transmission remains the predominant mode of HIV acquisition globally, accounting for the majority of new infections. Heterosexual transmission is particularly significant in sub-Saharan Africa, where cultural, socio-economic, and gender inequalities contribute to heightened vulnerability among women and adolescent girls^[7]^. MSM continue to be disproportionately affected by HIV/AIDS in many parts of the world, with elevated transmission risks compounded by social stigma, discrimination, and legal barriers to accessing healthcare services^[8]^. Injection drug use represents another critical pathway for HIV transmission, particularly in settings where injecting practices are prevalent and harm reduction services are inadequate or inaccessible^[9]^. The sharing of contaminated needles and syringes facilitates rapid HIV transmission within injecting drug user (IDU) networks, contributing to localized epidemics in urban centers and border regions. Efforts to expand access to sterile injecting equipment and opioid substitution therapy (OST) are essential components of comprehensive HIV prevention strategies for PWID^[10]^. Mother-to-child transmission (MTCT) remains a significant concern, albeit largely preventable with timely interventions. Effective PMTCT (prevention of mother-to-child transmission) programs, including antenatal screening, provision of ART to pregnant women living with HIV, and infant feeding counseling, have contributed to substantial reductions in new pediatric infections globally^[11]^. However, challenges persist in ensuring universal coverage of PMTCT services, particularly in resource-limited settings with weak health systems and limited access to maternal and child healthcare services. In recent years, there has been growing recognition of the syndemic nature of HIV/AIDS, characterized by intersecting epidemics of HIV and other infectious diseases, non-communicable diseases (NCDs), mental health disorders, and substance use disorders^[12]^. Co-infections with tuberculosis (TB), hepatitis B virus (HBV), and hepatitis C virus (HCV) present significant clinical challenges, particularly among people living with HIV who have compromised immune systems^[13]^. Integrated healthcare delivery models that address the holistic health needs of individuals living with HIV/AIDS are essential for optimizing health outcomes and reducing morbidity and mortality associated with co-infections. Emerging trends in HIV epidemiology include demographic shifts, such as aging populations among people living with HIV due to improved treatment outcomes and longer life expectancy. The aging HIV population faces unique challenges related to chronic comorbidities, mental health disorders, and social isolation, necessitating tailored healthcare services and support systems^[14]^. Additionally, behavioral factors, including changing sexual norms, migration patterns, and the influence of digital technologies on sexual networking and risk behavior, continue to shape HIV transmission dynamics in diverse settings^[15]^. The role of social determinants of health, including poverty, gender inequality, lack of education, and human rights violations, cannot be overstated in driving HIV/AIDS disparities. Structural barriers such as criminalization of key populations, discriminatory laws, and barriers to accessing healthcare services perpetuate vulnerability to HIV infection and hinder efforts to achieve epidemic control^[16]^. Addressing these underlying determinants through policy reform, advocacy for human rights, and community-led interventions is essential for advancing HIV prevention, treatment, and care agendas.

Addressing the immense global burden of HIV/AIDS requires not only innovative public health strategies but also substantial and sustained financial investment. The scale of the epidemic and its far-reaching implications highlight the critical role of major funders in supporting prevention, treatment, and care initiatives. These funding mechanisms form the backbone of the current funding landscape, ensuring that resources are allocated to mitigate the impact of HIV/AIDS globally. One of the most prominent funders in the fight against HIV/AIDS is the United States government, primarily through the PEPFAR. Launched in 2003, PEPFAR represents the largest commitment by any nation to combat a single disease. As of 2023, PEPFAR has invested over $100 billion in the global HIV/AIDS response, saving millions of lives and significantly reducing the incidence of new infections^[1]^. PEPFAR’s comprehensive approach includes funding for ART, prevention programs, testing and counseling services, and strengthening health systems in partner countries^[2]^. The program has also been instrumental in supporting innovative initiatives such as the DREAMS partnership, which aims to reduce HIV infections among adolescent girls and young women in sub-Saharan Africa^[3]^. The Global Fund to Fight AIDS, Tuberculosis, and Malaria is another critical player in the funding landscape. Established in 2002, the Global Fund is a partnership organization designed to accelerate the end of these three diseases. By pooling resources from governments, the private sector, and philanthropists, the Global Fund has disbursed more than $45 billion to support HIV/AIDS programs in over 100 countries^[4]^. The Global Fund’s model emphasizes country ownership, where recipient countries lead the design and implementation of programs tailored to their specific needs^[5]^. This approach has led to significant progress in increasing ART coverage, scaling up prevention efforts, and reducing HIV-related mortality^[6]^. The Bill & Melinda Gates Foundation is another major contributor, providing substantial financial support and advocacy for HIV/AIDS research and programs. Since its inception, the Foundation has committed over $3 billion to HIV/AIDS initiatives, focusing on vaccine development, biomedical prevention strategies, and improving access to care in low- and middle-income countries^[7]^. The Foundation’s investments have supported critical research leading to breakthroughs in HIV prevention technologies, such as PrEP and long-acting injectable ART^[8]^. Additionally, the Gates Foundation has played a key role in fostering PPPs and leveraging additional resources to amplify the impact of its funding^[9]^. UNAIDS, the UNAIDS, serves as the main advocate for global action against HIV/AIDS. Although not a direct funding agency, UNAIDS coordinates international efforts and mobilizes resources from various stakeholders to support national HIV/AIDS programs^[10]^. By setting global targets, such as the 90-90-90 goals, UNAIDS has provided a framework for countries to measure their progress in diagnosing, treating, and achieving viral suppression among people living with HIV^[11]^. The organization’s efforts in advocacy, policy guidance, and technical support have been pivotal in maintaining global commitment and funding for the HIV/AIDS response^[12]^. The World Bank has also been a significant funder, primarily through its Multi-Country HIV/AIDS Program (MAP) launched in 2000. The World Bank has committed over $4.6 billion to support HIV/AIDS programs in more than 30 countries, focusing on scaling up prevention, treatment, and care services^[13]^. The Bank’s approach emphasizes integrating HIV/AIDS interventions into broader health systems and addressing the socio-economic determinants of the epidemic^[14]^. Through its financial and technical assistance, the World Bank has helped countries build sustainable health infrastructure and capacity to respond to HIV/AIDS^[15]^. Several European countries have been major contributors to global HIV/AIDS funding. The United Kingdom, through its Department for International Development (DFID), has been one of the largest bilateral donors. The UK has committed over $2 billion to the Global Fund and has supported various bilateral HIV/AIDS programs in sub-Saharan Africa and other regions^[16]^. Germany, France, and the Netherlands have also provided significant financial support through contributions to the Global Fund and bilateral initiatives^[17]^. These countries have prioritized funding for comprehensive HIV/AIDS programs, including prevention, treatment, and health system strengthening^[18]^. In addition to governmental and multilateral organizations, private sector companies have increasingly become involved in funding HIV/AIDS initiatives. Pharmaceutical companies, such as Gilead Sciences, ViiV Healthcare, and Johnson & Johnson, have contributed not only through the development and distribution of antiretroviral drugs but also by supporting community-based programs and research initiatives^[19]^. These companies have engaged in PPPs to expand access to life-saving medications in low- and middle-income countries, often through tiered pricing strategies and voluntary licensing agreements^[20]^. Philanthropic organizations and individual donors have also played a crucial role. For example, the Elton John AIDS Foundation has raised and distributed over $450 million since its founding in 1992, supporting innovative programs focusing on marginalized and vulnerable populations^[21]^. Similarly, the Ford Foundation, the Rockefeller Foundation, and the Open Society Foundations have provided substantial funding for HIV/AIDS-related initiatives, particularly in human rights, advocacy, and policy reform^[22]^. The impact of these funding efforts has been profound. The scaling up of ART has averted millions of deaths and improved the quality of life for people living with HIV^[23]^. Prevention programs, including condom distribution, voluntary medical male circumcision, and harm reduction services, have significantly reduced new HIV infections^[24]^. Moreover, investments in strengthening the health system have enhanced the capacity of countries to deliver comprehensive HIV services and respond to other health challenges^[25]^. Despite these successes, challenges remain. Donor fatigue and shifting global priorities pose risks to sustained funding. Economic instability, exacerbated by the COVID-19 pandemic, has strained resources and diverted attention from HIV/AIDS to other urgent health issues^[26]^. Furthermore, stigma and discrimination continue to hinder access to services for key populations, such as MSM, sex workers, PWID, and transgender individuals^[27]^. To address these challenges, innovative financing mechanisms are essential. PPPs can leverage additional resources and expertise to support HIV/AIDS programs^[28]^. Social impact bonds and other results-based financing models can enhance the efficiency and impact of funding by tying financial incentives to achieve specific health outcomes^[29]^. Domestic resource mobilization is also critical, as countries must increasingly rely on their resources to sustain HIV/AIDS responses in the face of declining external aid^[30]^. Integrating HIV services with other health and social services can improve cost-effectiveness and ensure a holistic approach to healthcare. For example, integrating HIV testing and treatment with tuberculosis and sexual and reproductive health services can enhance the efficiency of service delivery and address the broader health needs of individuals^[31]^. Strengthening health information systems and using data-driven approaches can improve program planning, monitoring, and evaluation^[32]^. Community engagement and leadership are vital for the sustainability of HIV/AIDS programs. People living with HIV, key populations, and affected communities must be actively involved in the design, implementation, and evaluation of programs^[33]^. Their insights and experiences are crucial for developing interventions responsive to the needs and realities of those most affected by the epidemic^[34]^. Community-led initiatives have successfully reached marginalized populations and promoted human rights^[35]^. Advocacy and policy reform are also essential to create an enabling environment for effective HIV/AIDS responses. Legal and policy barriers, such as the criminalization of key populations and restrictive drug policies, must be addressed to reduce vulnerability to HIV and ensure equitable access to services^[36]^. Advocacy efforts should focus on promoting human rights, reducing stigma, and ensuring that HIV/AIDS remains a priority on the global health agenda^[37]^.

These financial contributions are channeled through various funding mechanisms that enable the implementation of targeted HIV/AIDS programs. Among these, grants are one of the most prevalent approaches, offering non-repayable funds provided by governments, multilateral organizations, foundations, and other entities to support specific projects or activities. Grants are crucial for funding various interventions, including ART, prevention programs, capacity building, and research. For instance, the Global Fund to Fight AIDS, Tuberculosis, and Malaria operates primarily through grants, disbursing billions of dollars to support national HIV/AIDS programs in low- and middle-income countries^[1]^. The advantage of grants is that they do not burden recipient countries with debt, allowing them to focus on implementing effective interventions. However, grants can be subject to donor conditions and may require extensive reporting and accountability measures^[2]^. Loans, while less common than grants in the context of HIV/AIDS funding are another important mechanism. These are funds provided to governments or organizations with the expectation of repayment, often with interest. The World Bank is a significant provider of loans for health-related projects, including HIV/AIDS initiatives. For example, the World Bank’s Multi-Country HIV/AIDS Program (MAP) has provided substantial loans to support HIV/AIDS responses in sub-Saharan Africa^[3]^. Loans can enable countries to scale up their health infrastructure and services quickly. However, the repayment obligations can strain national budgets, particularly in low-income countries, and may divert resources from other critical areas^[4]^. Donations from individuals, corporations, and philanthropic organizations are vital in HIV/AIDS funding. Philanthropic organizations, such as the Bill & Melinda Gates Foundation, the Elton John AIDS Foundation, and the Ford Foundation, have significantly contributed to the global HIV/AIDS response^[5]^. These donations often support innovative research, pilot projects, and interventions targeting marginalized populations. Corporate donations through corporate social responsibility (CSR) initiatives have also contributed to HIV/AIDS programs. For instance, pharmaceutical companies like Gilead Sciences and ViiV Healthcare have provided substantial funding and in-kind contributions to support treatment and prevention efforts^[6]^. Donations are typically flexible and can be directed to high-need areas, but they may be unpredictable and subject to the financial health and priorities of the donors^[7]^. Innovative financing mechanisms are increasingly being explored to address funding gaps and enhance the sustainability of HIV/AIDS programs. PPPs are a prominent example of how governments and private sector entities collaborate to leverage resources and expertise. These partnerships can mobilize additional funding, improve service delivery, and foster innovation. For instance, the Global HIV Vaccine Enterprise is a collaboration between the public and private sectors to accelerate the development of an HIV vaccine^[8]^. Similarly, PPPs have been instrumental in expanding access to ART. For example, the partnership between PEPFAR (the U.S. PEPFAR) and pharmaceutical companies has significantly reduced drug prices, allowing broader access in resource-limited settings^[10]^. The Global Fund to Fight AIDS, Tuberculosis, and Malaria, in collaboration with private corporations like Chevron, has co-funded HIV prevention programs, showcasing the ability of PPPs to amplify resources and outreach^[11]^. However, PPPs require strong governance structures and clear agreements to ensure the interests of all parties are aligned^[9]^. They also face challenges related to scalability, as their reliance on large-scale funding and infrastructure may need to be replicable in smaller or less resourceful settings. Accountability remains a concern, with critics noting potential conflicts between private sector profit motives and public health goals^[12]^.

Social impact bonds (SIBs) are another innovative financing approach gaining traction in the health sector, including HIV/AIDS. SIBs involve private investors providing upfront capital for social programs, with returns contingent on achieving specific outcomes. Governments or other entities repay the investors based on the success of the interventions. This results-based financing model can incentivize efficiency and effectiveness in program implementation. For example, the world’s first development impact bond in health, launched by the International Committee of the Red Cross, aims to improve HIV outcomes in Kenya and Uganda^[10]^. In South Africa, a pilot SIB aimed at reducing mother-to-child transmission of HIV demonstrated the model’s potential to align financial incentives with health outcomes^[14]^. While SIBs have the potential to attract new sources of funding, they require rigorous evaluation frameworks and can be complex to design and manage^[11]^. Challenges such as high transaction costs and reliance on robust data systems limit their scalability in resource-constrained settings^[15]^.

Comparatively, PPPs excel in mobilizing large-scale resources and leveraging expertise from multiple sectors, making them ideal for comprehensive, multi-faceted interventions. However, their success often depends on effective governance structures and alignment of stakeholder interests. SIBs, while smaller in scale, provide a results-oriented approach that fosters innovation and accountability. Integrating these mechanisms could address their respective limitations. For example, PPPs could fund infrastructure and capacity-building initiatives. At the same time, SIBs focus on specific, measurable outcomes within these programs, creating a hybrid model that leverages the strengths of both approaches. Such integration would require strong governance frameworks to ensure alignment and coherence. Blended finance, which combines public and private funding, is another approach to support HIV/AIDS initiatives. By leveraging concessional finance from donors to attract private sector investment, blended finance can mobilize larger amounts of capital for HIV/AIDS programs. This approach can enhance the impact of limited public resources and foster innovation. The Health Impact Fund, proposed by the Incentives for Global Health, is an example of a blended finance mechanism that incentivizes pharmaceutical companies to develop and provide affordable treatments for diseases like HIV/AIDS^[14]^. Blended finance requires careful structuring to align the interests of all stakeholders and ensure that public resources are used effectively^[15]^. Global health initiatives and multilateral organizations play a crucial role in coordinating and channeling funding for HIV/AIDS. As previously mentioned, the Global Fund pools resources from various donors to support HIV/AIDS, tuberculosis, and malaria programs. The United States PEPFAR is another major initiative, providing substantial funding and technical assistance to countries heavily affected by HIV/AIDS^[16]^. These initiatives have significantly contributed to scaling up HIV services and achieving global health targets. However, they also face challenges related to sustainability, donor dependency, and the need for increased domestic investment^[17]^. Crowdfunding and digital fundraising platforms have emerged as innovative ways to mobilize resources for HIV/AIDS programs. Platforms like GlobalGiving and GoFundMe enable individuals and organizations to raise funds for specific projects and interventions. Crowdfunding can engage a broad base of supporters and provide flexible funding for community-based initiatives. For example, numerous HIV/AIDS organizations have successfully raised funds through these platforms to support prevention and care services^[18]^. While crowdfunding can generate significant resources, attracting donors requires effective communication and outreach strategies^[19]^. Conditional cash transfers (CCTs) and other incentives-based funding mechanisms are also being explored to enhance the impact of HIV/AIDS programs. CCTs provide financial incentives to individuals or households contingent on meeting certain health-related conditions, such as attending HIV testing and counseling sessions or adhering to ART. These mechanisms can improve health-seeking behaviors and outcomes among vulnerable populations. For instance, studies in sub-Saharan Africa have shown that CCTs can increase the uptake of HIV testing and reduce risky behaviors^[20]^. However, CCT programs must be carefully designed to ensure sustainability and avoid unintended consequences^[21]^. Health insurance schemes and social health protection programs are vital for ensuring that people living with HIV have access to affordable and comprehensive care. Integrating HIV services into national health insurance schemes can enhance financial protection and reduce out-of-pocket expenses for patients. Countries like Rwanda and Thailand have successfully integrated HIV services into their national health insurance programs, improving access to treatment and care^[22]^. Expanding health insurance coverage and ensuring the inclusion of HIV services require strong political commitment and adequate financing^[23]^. Foundations and international non-governmental organizations (NGOs) also play a significant role in funding HIV/AIDS initiatives. Organizations like the Clinton Health Access Initiative (CHAI) and the Elizabeth Glaser Pediatric AIDS Foundation (EGPAF) have provided substantial funding and technical support to scale up HIV services and improve health outcomes^[24]^. These organizations often focus on innovative approaches, capacity building, and addressing gaps in service delivery. The involvement of foundations and NGOs is crucial for reaching marginalized populations and advocating for policy changes^[25]^. Regional funding mechanisms, such as the African Union’s AIDS Watch Africa (AWA) and the Caribbean Community’s Pan Caribbean Partnership Against HIV/AIDS (PANCAP), also contribute to the HIV/AIDS response. These mechanisms facilitate regional cooperation, mobilize resources, and support the implementation of regional strategies and programs. AWA, for instance, has played a key role in advocating for increased domestic investment in HIV/AIDS and supporting the implementation of the African Union’s Catalytic Framework to End AIDS, TB, and Malaria by 2030^[26]^. Regional mechanisms enhance coordination and collaboration among countries but require sustained political and financial commitment^[27]^. Traditional bilateral aid from high-income countries remains a significant source of funding for HIV/AIDS programs. Countries like the United Kingdom, France, Germany, and the Netherlands have provided substantial bilateral aid to support HIV/AIDS initiatives in low- and middle-income countries^[28]^. Bilateral aid often focuses on specific regions or countries, aligning with the donor’s strategic priorities. For example, the UK’s Department for International Development (DFID) has supported HIV/AIDS programs in sub-Saharan Africa and South Asia^[29]^. Bilateral aid can provide targeted support, but it may be influenced by geopolitical considerations and donor priorities^[30]^. Debt relief initiatives and debt swaps are innovative mechanisms that can free up resources for HIV/AIDS programs in heavily indebted countries. Debt relief initiatives, such as the Heavily Indebted Poor Countries (HIPC) Initiative, aim to reduce the debt burden of eligible countries, enabling them to allocate more resources to health and development programs^[31]^. Debt swaps involve creditors forgiving debt in exchange for the debtor country investing the equivalent amount in health or social programs. For example, debt swaps have been used to fund HIV/AIDS programs in countries like Mozambique and Zambia^[32]^. These mechanisms can provide significant funding but require coordination and agreement between creditors and debtor countries^[33]^.

Over time, funding mechanisms such as grants have reflected broader trends in the global response to HIV/AIDS. In the early 2000s, this response witnessed unprecedented levels of financial support, driven by the recognition of the epidemic as a major public health crisis. The establishment of the Global Fund to Fight AIDS, Tuberculosis, and Malaria in 2002 and the launch of the U.S. PEPFAR in 2003 marked a significant increase in international funding for HIV/AIDS^[1,2]^. These initiatives mobilized billions of dollars, enabling countries to scale up prevention, treatment, and care services. Between 2000 and 2010, global funding for HIV/AIDS increased dramatically, with contributions from bilateral donors, multilateral organizations, and philanthropic foundations^[3]^. However, funding trends have experienced notable shifts in recent years. One significant trend is the plateauing of international funding for HIV/AIDS. After peaking around 2013, global funding levels have largely stagnated or even declined in some cases^[4]^. According to the UNAIDS Global AIDS Update 2020, total international resources available for HIV in 2019 were approximately $19.8 billion, falling short of the estimated $26.2 billion needed to achieve global targets^[5]^. This stagnation is partly due to changing donor priorities, economic constraints, and competing health and development challenges. Several factors have contributed to the shifting priorities of international donors. The global economic crisis of 2008-2009 had a lasting impact on the fiscal capacities of donor countries, leading to budget cuts and reallocations of aid resources^[6]^. Additionally, the emergence of other global health threats, such as Ebola, Zika, and, more recently, the COVID-19 pandemic, has diverted attention and resources away from HIV/AIDS^[7]^. The COVID-19 pandemic, in particular, has strained health systems and budgets worldwide, causing disruptions in HIV services and funding^[8]^.

Another notable trend is the increasing emphasis on domestic resource mobilization for HIV/AIDS. Recognizing the need for sustainable financing, many countries have increased their domestic investments in HIV/AIDS programs. For example, South Africa has significantly ramped up its domestic funding, becoming one of the largest funders of its national HIV response^[9]^. Domestic resource mobilization is seen as critical for reducing dependency on external aid and ensuring the long-term sustainability of HIV/AIDS programs. However, the capacity to mobilize domestic resources varies widely among countries, with low-income countries facing significant challenges^[10]^. Philanthropic funding has also played a crucial role in supporting HIV/AIDS initiatives, and trends in this sector have evolved. Major foundations such as the Bill & Melinda Gates Foundation, the Elton John AIDS Foundation, and the Ford Foundation have continued to provide substantial funding for HIV/AIDS research, prevention, and treatment programs^[11-13]^. Philanthropic contributions have been instrumental in advancing innovative approaches and addressing gaps in funding. However, reliance on philanthropic funding can be unpredictable, as it is often influenced by the priorities and financial health of the donors^[14]^. Innovative financing mechanisms have gained traction as a means to address funding gaps and enhance the sustainability of HIV/AIDS programs. Examples of such mechanisms include PPPs, social impact bonds (SIBs), and blended finance. PPPs leverage the resources and expertise of the private sector to support HIV/AIDS programs. For instance, the Global HIV Vaccine Enterprise is a collaboration between the public and private sectors to accelerate the development of an HIV vaccine^[15]^. SIBs, which involve private investors providing upfront capital for social programs with returns contingent on achieving specific outcomes, are being explored to fund HIV/AIDS interventions^[16]^. Blended finance, which combines public and private funding, is also used to mobilize capital for HIV/AIDS programs^[17]^. The role of multilateral organizations in funding HIV/AIDS has also seen changes. The Global Fund remains a cornerstone of the global HIV/AIDS response, but it has faced funding challenges. In recent replenishment cycles, the Global Fund has set ambitious targets to mobilize resources, yet meeting these targets has been difficult due to donor fatigue and economic constraints^[18]^. The World Bank and regional development banks have continued to provide financial and technical support, but their focus has increasingly shifted towards broader health system strengthening and integrated health services^[19]^. Donor funding for HIV/AIDS has also been influenced by the global push for universal health coverage (UHC). Integrating HIV services into broader health systems and UHC frameworks has become a priority for many countries and donors. This approach ensures that HIV services are accessible, affordable, and sustainable within health care^[20]^. However, integrating HIV services into UHC requires significant investment in health systems and may pose challenges in maintaining the focus and quality of HIV-specific interventions^[21]^. Emerging economies and middle-income countries are playing an increasingly important role in funding HIV/AIDS programs, both domestically and internationally. Countries like Brazil, China, and India have expanded their domestic HIV responses and contributed to regional and global initiatives^[22]^. South-South cooperation, where developing countries share knowledge, resources, and technology, has become a valuable mechanism for strengthening HIV responses in the Global South^[23]^. This trend reflects a shift towards greater ownership and leadership by affected countries in addressing their HIV epidemics. The landscape of funding for HIV/AIDS research has also evolved. Significant investments have been made in biomedical research, leading to advancements in HIV treatment and prevention technologies, such as PrEP, long-acting antiretroviral drugs, and potential vaccines^[24]^. However, funding for HIV research faces challenges, including competition with other research priorities and the need for sustained investment to achieve breakthroughs^[25]^. The COVID-19 pandemic has further highlighted the importance of resilient research funding mechanisms that can adapt to emerging health threats^[26]^. Human rights and advocacy organizations have continued to play a critical role in mobilizing funding and support for HIV/AIDS. Advocacy efforts have focused on ensuring that funding is directed towards evidence-based interventions, addressing stigma and discrimination, and promoting the rights of key populations affected by HIV^[27]^. The Global Fund’s Community, Rights, and Gender (CRG) strategic initiative is an example of efforts to ensure that funding addresses the needs and rights of vulnerable populations^[28]^. Advocacy organizations have also held governments and donors accountable for their commitment to HIV funding^[29]^. Regional and national HIV funding mechanisms have seen significant developments. For example, the African Union’s AIDS Watch Africa (AWA) and the Caribbean Community’s Pan Caribbean Partnership Against HIV/AIDS (PANCAP) have facilitated regional cooperation and resource mobilization^[30]^. These mechanisms support the implementation of regional strategies and programs, enhancing coordination and collaboration among countries^[31]^. Regional approaches are crucial for addressing cross-border issues and ensuring a comprehensive response to the epidemic^[32]^. Crowdfunding and digital fundraising platforms have emerged as innovative ways to mobilize resources for HIV/AIDS programs. Platforms like GlobalGiving and GoFundMe enable individuals and organizations to raise funds for specific projects and interventions^[33]^. Crowdfunding can engage a broad base of supporters and provide flexible funding for community-based initiatives. However, the success of crowdfunding campaigns often depends on effective communication and outreach strategies to attract donors^[34]^. Conditional cash transfers (CCTs) and other incentives-based funding mechanisms are being explored to enhance the impact of HIV/AIDS programs. CCTs provide financial incentives to individuals or households contingent on meeting certain health-related conditions, such as attending HIV testing and counseling sessions or adhering to ART^[35]^. These mechanisms can improve health-seeking behaviors and outcomes among vulnerable populations. Studies in sub-Saharan Africa have shown that CCTs can increase the uptake of HIV testing and reduce risky behaviors^[36]^. However, CCT programs must be carefully designed to ensure sustainability and avoid unintended consequences^[37]^. Health insurance schemes and social health protection programs are vital for ensuring that people living with HIV have access to affordable and comprehensive care. Integrating HIV services into national health insurance schemes can enhance financial protection and reduce out-of-pocket patient expenses^[38]^. Countries like Rwanda and Thailand have successfully integrated HIV services into their national health insurance programs, improving access to treatment and care^[39]^. Expanding health insurance coverage and ensuring the inclusion of HIV services require strong political commitment and adequate financing^[40]^. Foundations and international NGOs have continued to play a significant role in funding HIV/AIDS initiatives. Organizations like the Clinton Health Access Initiative (CHAI) and the Elizabeth Glaser Pediatric AIDS Foundation (EGPAF) have provided substantial funding and technical support to scale up HIV services and improve health outcomes^[41]^. These organizations often focus on innovative approaches, capacity building, and addressing gaps in service delivery. The involvement of foundations and NGOs is crucial for reaching marginalized populations and advocating for policy changes^[42]^. Debt relief initiatives and debt swaps are innovative mechanisms that can free up resources for HIV/AIDS programs in heavily indebted countries. Debt relief initiatives, such as the Heavily Indebted Poor Countries (HIPC) Initiative, aim to reduce the debt burden of eligible countries, enabling them to allocate more resources to health and development programs^[43]^. Debt swaps involve creditors forgiving debt in exchange for the debtor country investing the equivalent amount in health or social programs. For example, debt swaps have been used to fund HIV/AIDS programs in countries like Mozambique and Zambia^[44]^. These mechanisms can provide significant funding but require coordination and agreement between creditors and debtor countries^[45]^. Traditional bilateral aid from high-income countries remains a significant source of funding for HIV/AIDS programs. Countries like the United Kingdom, France, Germany, and the Netherlands have provided substantial bilateral aid to support HIV/AIDS initiatives in low- and middle-income countries^[46]^. Bilateral aid often focuses on specific regions or countries, aligning with the donor’s strategic priorities. For example, the UK’s Department for International Development (DFID) has supported HIV/AIDS programs in sub-Saharan Africa and South Asia^[47]^. Bilateral aid can provide targeted support, but it may be influenced by geopolitical considerations and donor priorities, leading to fluctuations in funding levels and focus areas^[48]^. One of the emerging trends in HIV/AIDS funding is the increased focus on integrated health services. This approach seeks to provide comprehensive health care that includes HIV services as part of a broader package of health interventions. Integrated health services can improve health outcomes by addressing comorbidities such as tuberculosis, hepatitis, and non-communicable diseases (NCDs) alongside HIV. This holistic approach can enhance efficiency and resource utilization. For example, integrating HIV services with maternal and child health programs can improve outcomes for both mothers and children^[49]^. However, implementing integrated services requires substantial investment in health systems strengthening and coordination among various health programs^[50]^. The role of civil society organizations (CSOs) and community-based organizations (CBOs) in HIV/AIDS funding has gained prominence. These organizations are critical for reaching key populations, advocating for policy changes, and implementing grassroots interventions. Many international donors and funding agencies have recognized the importance of supporting CSOs and CBOs to enhance the effectiveness of HIV responses. For instance, the Global Fund’s Community, Rights, and Gender (CRG) initiative emphasizes the importance of community-led responses and provides funding to CSOs to support their work^[51]^. Ensuring sustainable funding for these organizations is essential for maintaining the momentum of community-based HIV initiatives^[52]^. Digital health technologies have also influenced funding trends in HIV/AIDS. The adoption of mobile health (mHealth) and digital platforms has facilitated the delivery of HIV services, including education, prevention, testing, and adherence support. These technologies have the potential to reach underserved populations and improve the efficiency of service delivery. Donors and funding agencies increasingly invest in digital health solutions to enhance HIV responses. For example, PEPFAR has supported developing and implementing digital health interventions to improve HIV outcomes^[53]^. However, scaling up digital health solutions requires investment in infrastructure, capacity building, and addressing issues related to data security and privacy^[54]^. In recent years, there has been a growing emphasis on addressing the social determinants of health in HIV/AIDS funding. Social determinants such as poverty, education, gender inequality, and stigma significantly impact HIV vulnerability and health outcomes. Donors and funding agencies increasingly recognize the importance of addressing these underlying factors to achieve sustainable HIV responses. For example, the Global Fund’s funding model focuses on gender and human rights to ensure that interventions address the root causes of HIV vulnerability^[55]^. Addressing social determinants requires a multisectoral approach and collaboration across various sectors, including education, social protection, and gender equality^[56]^. The private sector’s involvement in HIV/AIDS funding has expanded, with corporate social responsibility (CSR) initiatives and private-public partnerships playing a significant role. Pharmaceutical companies, in particular, have contributed to HIV/AIDS funding through donations of antiretroviral drugs, support for research and development, and funding for prevention and treatment programs. Companies like Gilead Sciences and ViiV Healthcare have substantially invested in HIV/AIDS initiatives^[57]^. CSR initiatives can complement public funding and bring additional resources to the HIV response. However, the sustainability and alignment of private sector contributions with public health goals remain critical^[58]^. Trends in HIV/AIDS funding have also been influenced by the increasing recognition of key populations’ needs. Key populations, including MSM, sex workers, PWID, and transgender individuals, are disproportionately affected by HIV and often face barriers to accessing services. Funding agencies and donors emphasize targeted interventions for key populations to reduce disparities and improve health outcomes. The Global Fund and PEPFAR have specific strategies and funding allocations to address the needs of key populations^[59]^. Ensuring that funding reaches key populations requires inclusive policies, stigma reduction efforts, and community engagement^[60]^. International funding for HIV/AIDS has seen a shift towards more performance-based funding models. These models tie funding disbursements to achieving specific targets and outcomes, incentivizing efficiency and effectiveness in program implementation. The Global Fund’s New Funding Model (NFM) incorporates performance-based funding principles, rewarding countries for achieving results and improving health outcomes^[61]^. Performance-based funding can enhance accountability and drive improvements in service delivery. However, it also requires robust monitoring and evaluation systems to measure progress and outcomes^[62]^ accurately. The sustainability of HIV/AIDS funding remains a critical concern as international donors re-evaluate their commitments. The transition from donor-funded programs to domestically funded and managed initiatives is a complex process that requires careful planning and capacity building. Countries transitioning from donor support must ensure that there are no disruptions in HIV services and that gains made over the years are sustained. This transition also involves addressing challenges related to governance, health financing, and service delivery^[63]^. The UNAIDS has been supporting countries in their transition planning to ensure sustainable HIV responses^[64]^. Regional disparities in HIV/AIDS funding continue to pose challenges. Sub-Saharan Africa, which bears the highest burden of HIV, receives a significant proportion of international HIV funding. However, other regions, such as Eastern Europe and Central Asia, have seen declining funding levels despite rising HIV incidence rates^[65]^. Addressing regional disparities requires a balanced approach to resource allocation that considers different regions’ epidemiological contexts and funding needs. Ensuring equitable access to funding is essential for achieving global HIV targets and reducing disparities in health outcomes^[66]^.

### Challenges in HIV/AIDS funding

Sustainable funding for HIV/AIDS initiatives is paramount in the global effort to combat one of the most significant public health challenges of our time. Since the emergence of HIV and AIDS in the early 1980s, the epidemic has exacted a profound toll on individuals, communities, and healthcare systems worldwide^[1]^. Over the decades, concerted efforts have been made to expand access to prevention, treatment, and care services, resulting in remarkable advancements in reducing AIDS-related morbidity and mortality^[2]^. However, the sustainability of these gains hinges crucially on secure and predictable funding streams that can withstand economic fluctuations, shifting political priorities, and evolving epidemiological trends. The financial resources required to address HIV/AIDS effectively are substantial and multifaceted. Funding supports a wide array of interventions, including HIV testing and counseling, ART, PMTCT programs, condom distribution, harm reduction services for PWID, and community-based outreach efforts^[3]^. These interventions are essential not only for saving lives but also for preventing new infections and reducing the overall burden of the disease on healthcare systems and economies. Historically, international funding for HIV/AIDS has been a cornerstone of the global response. Initiatives such as the Global Fund to Fight AIDS, Tuberculosis, and Malaria, PEPFAR, and bilateral aid from donor countries have played pivotal roles in mobilizing resources and supporting country-led efforts to scale up HIV/AIDS services^[4,5]^. These funding mechanisms have catalyzed progress in expanding access to ART, strengthening healthcare infrastructure, and promoting evidence-based prevention strategies in low- and middle-income countries disproportionately affected by the epidemic. The economic rationale for sustained HIV/AIDS funding is compelling. Investments in HIV/AIDS programs yield significant returns by averting future healthcare costs, boosting workforce productivity, and mitigating the broader socio-economic impacts of the epidemic^[6]^. Studies have shown that every dollar invested in HIV prevention and treatment yields substantial health benefits and economic returns, underscoring the cost-effectiveness of targeted interventions^[7]^. Moreover, sustained funding fosters innovation in biomedical research, pharmaceutical development, and health system strengthening, driving continuous improvements in HIV/AIDS care and management. Beyond economic considerations, sustainable funding is essential for upholding human rights and promoting social justice. Access to HIV/AIDS services is a fundamental right enshrined in international declarations and conventions^[8]^. Sustainable funding ensures that marginalized and vulnerable populations, including women, children, adolescents, sex workers, PWID, and MSM, have equitable access to life-saving interventions without discrimination or stigma^[9]^. Moreover, robust financial support enables communities affected by HIV/AIDS to actively participate in decision-making processes, advocate for their rights, and contribute to shaping responsive and inclusive healthcare systems. The global response to HIV/AIDS has made significant strides, evidenced by the dramatic expansion of ART coverage and reductions in new HIV infections and AIDS-related deaths in many parts of the world^[10]^. However, progress is uneven, with stark disparities within and between countries. Sustainable funding is essential for addressing these inequities and closing the gaps in access to HIV/AIDS services, particularly in regions where health systems are fragile, resources are limited, and the burden of disease remains disproportionately high^[11]^. Political commitment to HIV/AIDS funding is a critical determinant of sustainability. Governments, donor agencies, multilateral organizations, and private sector partners must honor financial pledges, uphold transparency in resource allocation, and prioritize long-term investments in health systems strengthening^[12]^. Advocacy efforts by civil society organizations, people living with HIV/AIDS (PLWHA), and community leaders play a crucial role in holding stakeholders accountable and ensuring that HIV/AIDS remains a global health priority. Looking ahead, the goal of ending the HIV/AIDS epidemic by 2030, as outlined in the SDGs, hinges on sustained political will and financial commitment^[13]^. Achieving this ambitious target requires innovative financing mechanisms, strategic partnerships, and evidence-based approaches tailored to the epidemiological context of each country. Lessons learned from successful funding models and best practices in resource mobilization can inform future strategies for maximizing the impact of investments in HIV/AIDS prevention, treatment, and care.

Government funding is a key type of support for the effective response to HIV/AIDS. Governments allocate funds through national budgets to support a wide range of HIV/AIDS-related activities, including prevention programs, treatment and care services, research and development, and community support initiatives. This funding is critical for implementing national HIV/AIDS strategies, ensuring that resources are directed toward the most effective and necessary interventions. By providing financial support, governments play a pivotal role in combating the epidemic, reducing new infections, and improving the quality of life for PLWHA. Table 2 highlights the diverse sources of HIV/AIDS funding, illustrating how different sectors contribute to the fight against HIV/AIDS through various mechanisms and facing distinct challenges. The examples provide real-world contexts for these funding types, showcasing successful initiatives and partnerships. One of the key areas where government funding is directed is prevention programs. These programs are designed to reduce the transmission of HIV through education, awareness campaigns, and the distribution of prevention tools such as condoms and clean needles. Government-funded prevention initiatives often include public health campaigns that promote safe sex practices, regular HIV testing, and the reduction of stigma and discrimination associated with the virus. Additionally, governments may fund needle exchange programs and harm reduction services for PWID, which are highly effective in preventing HIV transmission^[67]^. Treatment and care services are another critical component of government-funded HIV/AIDS programs. Access to ART is essential for managing HIV infection, improving health outcomes, and reducing the risk of transmission. Government funding supports the procurement and distribution of ART, ensuring it is available and affordable for all who need it. In many countries, national health programs cover the cost of ART, making it accessible to people regardless of income level. Government funding also supports healthcare infrastructure, including clinics and hospitals that provide comprehensive HIV care and training for healthcare workers to ensure they can deliver high-quality services^[68]^. Research and development (R&D) is a crucial aspect of government funding for HIV/AIDS. Investing in R&D enables the discovery of new treatments, vaccines, and diagnostic tools that can improve the management of HIV and ultimately lead to a cure. Governments allocate funds to public health research institutions, universities, and private sector partnerships to advance scientific knowledge and innovation in HIV/AIDS. These investments have led to significant breakthroughs, such as the development of more effective ART regimens and PrEP, which can prevent HIV infection in high-risk populations. By funding R&D, governments play a vital role in accelerating progress towards ending the HIV epidemic^[69]^. Community support initiatives are also a major focus of government funding for HIV/AIDS. These initiatives aim to address the social and economic determinants of health that contribute to the spread of HIV and affect the well-being of PLWHA. Government-funded programs may include support for housing, food security, mental health services, and employment assistance for individuals affected by HIV. Governments often fund NGOs and community-based organizations (CBOs) that provide essential services and advocacy for PLWHA. These organizations are often deeply embedded in their communities and can effectively reach and support marginalized and vulnerable populations^[70]^. The allocation of government funding for HIV/AIDS is guided by national HIV/AIDS strategies and plans. These strategies are typically developed through a collaborative process involving government agencies, healthcare providers, civil society organizations, and affected communities. National strategies outline the goals, priorities, and targets for the country’s HIV/AIDS response, ensuring that resources are directed toward the most impactful interventions. Governments use these strategies to coordinate efforts, monitor progress, and evaluate the effectiveness of funded programs. This strategic approach helps ensure government funding is used efficiently and achieves the desired outcomes^[71]^. Despite the significant contributions of government funding to the HIV/AIDS response, several challenges need to be addressed. One major challenge is the sustainability of funding. Many countries, especially those with limited resources, struggle to maintain consistent and adequate funding for HIV/AIDS programs. Economic downturns, shifts in political priorities, and competing health needs can all impact the availability of funds. To address this, governments must explore innovative financing mechanisms, such as PPPs and domestic resource mobilization, to ensure sustainable funding for HIV/AIDS initiatives^[72]^. Another challenge is the equitable distribution of funds. Ensuring that government funding reaches the populations most in need, including marginalized and vulnerable groups, requires careful planning and monitoring. In some cases, funding may be concentrated in urban areas, leaving rural and remote communities underserved. Governments must use data-driven approaches to identify and prioritize funding for high-burden areas and populations to achieve equity. Additionally, involving affected communities in decision-making processes can help ensure that funding addresses the real needs and challenges faced by those most impacted by HIV/AIDS^[73]^. Transparency and accountability are critical to the effective use of government funding for HIV/AIDS. Governments must establish clear and transparent processes for allocating, disbursing, and reporting on using funds. This includes establishing robust monitoring and evaluation systems to track funded programs’ impact and identify improvement areas. Public accountability mechanisms, such as regular audits and public reporting, help build trust among stakeholders and ensure that funds are used effectively and ethically. By maintaining transparency and accountability, governments can enhance the credibility and legitimacy of their HIV/AIDS programs^[8]^. Ethical considerations are paramount in government funding for HIV/AIDS. Ethical principles such as equity, justice, and respect for human rights should guide all funding decisions and program implementations. This means prioritizing interventions that reduce health disparities, protect the rights and dignity of PLWHA, and address the social determinants of health. Governments must ensure that funded programs do not contribute to stigma or discrimination and promote inclusive and non-coercive approaches. Upholding ethical standards in funding practices helps ensure that HIV/AIDS programs are not only effective but also just and humane^[9]^. International collaboration is another important aspect of government funding for HIV/AIDS. Many countries rely on international funding and technical assistance to support their HIV/AIDS programs. Global initiatives such as the Global Fund to Fight AIDS, Tuberculosis and Malaria and the PEPFAR provide substantial financial support to low- and middle-income countries. These initiatives complement national funding efforts and help scale up critical HIV/AIDS interventions. Governments must collaborate with international partners to align their funding strategies, share best practices, and leverage resources for maximum impact^[10]^.

**Table 2:** Types of HIV/AIDS funding, their sources, key contributors, mechanisms, advantages, challenges, and examples

Type of funding	Sources	Key contributors	Mechanisms	Advantages	Challenges	Examples
Government Funding	National governments, international bodies	US, UK, EU, Global Fund, PEPFAR	Tax revenue, international aid, grants	Large-scale funding, policy influence	Political instability, changing priorities, budget cuts	PEPFAR, Global Fund to Fight AIDS, Tuberculosis and Malaria
Private Sector Funding	Corporations, philanthropic foundations	Gilead, Johnson & Johnson, Gates Foundation	Corporate social responsibility, donations	Innovation, substantial resources, strategic investments	Profit motives, sustainability, alignment with public health goals	Gates Foundation’s HIV initiatives
Non-Governmental Organizations (NGOs)	Charitable donations, grants, international aid	MSF, Red Cross, local NGOs	Fundraising, donor campaigns, grants	Community engagement, flexibility, grassroots reach	Limited funds, dependency on donations, variability	Médecins Sans Frontières (MSF), International HIV/AIDS Alliance
Public-Private Partnerships (PPPs)	Combined resources from public and private sectors	Global Fund, UNITAID, Clinton Health Access Initiative	Co-investment, joint ventures, shared initiatives	Synergistic use of resources, innovation, scalability	Coordination complexities, accountability, differing priorities	UNITAID’s collaboration with pharmaceutical companies

The table presents the diverse types of funding mechanisms crucial for addressing the challenges of HIV/AIDS globally. Government funding, sourced from national budgets and international aid, plays a pivotal role due to its scale and policy influence, although it faces challenges like political instability and changing priorities. Private sector funding, driven by corporations and philanthropic foundations, brings innovation and substantial resources but must balance profit motives with public health goals. Non-governmental organizations (NGOs) rely on donations and grants and excel in community engagement and flexibility but need help with sustainability and funding variability. Public-private partnerships (PPPs) combine resources from both sectors, offering synergies in funding and innovation, yet face complexities in coordination and aligning priorities. Together, these funding types underscore the collaborative efforts required to combat HIV/AIDS effectively, each bringing unique strengths and challenges to the global response. [Authors’ creations].

Another important type of funding is from the private sector, which plays a crucial role in filling gaps left by public and international funding. Governments and international organizations often face budget constraints and competing priorities that limit their ability to address the needs of HIV/AIDS programs fully. The private sector can provide additional financial resources that enhance the scope and reach of these programs. For instance, private foundations such as the Bill & Melinda Gates Foundation have committed substantial funds to HIV/AIDS research, prevention, and treatment initiatives, significantly advancing the global response to the epidemic^[1]^. Corporate philanthropy, through direct donations and the establishment of dedicated HIV/AIDS programs, also contributes to the overall pool of resources available for combating HIV/AIDS. Innovation is a hallmark of private sector involvement in HIV/AIDS funding. Corporations, particularly those in the pharmaceutical and biotechnology industries, invest heavily in research and development (R&D) to discover new treatments, diagnostics, and preventive measures. These investments have led to the development of life-saving antiretroviral drugs, PrEP, and other biomedical innovations that have transformed HIV/AIDS care. The private sector’s emphasis on innovation extends to using technology and data analytics to improve program delivery and outcomes. For example, mobile health (mHealth) initiatives funded by private companies provide health information, support treatment adherence, and facilitate remote consultations for PLWHA^[2]^. PPPs are a key strategy for leveraging private sector resources and expertise. These partnerships bring together governments, NGOs, and private entities to address the HIV/AIDS epidemic collaboratively. PPPs can enhance the efficiency and effectiveness of HIV/AIDS programs by combining the strengths of each partner. For instance, the Global Fund to Fight AIDS, Tuberculosis, and Malaria has successfully mobilized private sector contributions through partnerships with corporations and foundations, significantly expanding its reach and impact^[3]^. Such collaborations facilitate knowledge sharing, innovation, and resource mobilization, leading to more comprehensive and sustainable HIV/AIDS interventions. Corporate social responsibility (CSR) drives many private sector contributions to HIV/AIDS funding. Companies recognize the importance of addressing public health issues, including HIV/AIDS, as part of their commitment to social responsibility and ethical business practices. CSR initiatives often focus on supporting employees, customers, and communities affected by HIV/AIDS. For example, many companies implement workplace programs that provide HIV testing, counseling, and treatment for their employees, reducing stigma and promoting health and well-being. Additionally, companies may support community-based programs offering local populations education, prevention, and care services^[4]^. Philanthropic foundations are significant contributors to HIV/AIDS funding. Foundations such as the Bill & Melinda Gates Foundation, the Elton John AIDS Foundation, and the Ford Foundation have made substantial financial commitments to HIV/AIDS initiatives. These foundations fund various activities, including research, advocacy, prevention, treatment, and support services. Philanthropic funding often targets innovative and high-impact projects that may not receive adequate support from other sources. For example, the Gates Foundation has invested in cutting-edge research to develop an HIV vaccine and has supported large-scale prevention programs in high-burden countries^[5]^. Despite the many benefits of private sector funding, challenges, and ethical considerations must be addressed. One challenge is ensuring the alignment of private sector initiatives with national HIV/AIDS strategies and priorities. Private sector programs should complement and enhance public health efforts rather than duplicate or undermine them. Effective coordination and collaboration between the private sector and public health authorities are essential to achieve this alignment. Additionally, transparency and accountability are crucial to ensure that private sector funds are used effectively and ethically. Companies and foundations must establish clear mechanisms for reporting and evaluating the impact of their HIV/AIDS initiatives^[6]^. Equity is an important ethical consideration in private-sector funding for HIV/AIDS. Private sector contributions must reach the populations most in need, including marginalized and vulnerable groups who often face barriers to accessing HIV/AIDS services. Private sector programs should prioritize equity by targeting high-burden areas and populations and by addressing the social determinants of health that contribute to HIV vulnerability. Involving affected communities in the design and implementation of programs can help ensure that interventions are culturally appropriate and responsive to local needs^[7]^. Intellectual property (IP) and access to medicines are critical ethical issues in the context of private-sector funding for HIV/AIDS. Pharmaceutical companies hold patents on many HIV/AIDS drugs, which can limit access to affordable treatment in low- and middle-income countries. Efforts to balance IP rights with public health needs have led to initiatives such as voluntary licensing agreements and the establishment of the Medicines Patent Pool, which facilitates the production of generic versions of patented drugs. These initiatives aim to increase the availability of affordable HIV/AIDS medications while respecting the rights of innovators^[8]^. Sustainability is another key concern in private-sector funding for HIV/AIDS. While private sector contributions can provide significant short-term support, it is important to ensure the long-term sustainability of HIV/AIDS programs. This requires integrating private sector initiatives into broader health systems and promoting domestic resource mobilization to sustain programs over time. PPPs and innovative financing mechanisms, such as social impact bonds and blended finance, can help achieve sustainable funding models by leveraging private investment for long-term impact^[9]^.

NGOs represent another type of funding source, operating independently of government control to serve the public interest through advocacy, service delivery, and community mobilization. Their origins can be traced back to early humanitarian movements and advocacy groups that emerged in response to social injustices and humanitarian crises^[10]^. Today, NGOs encompass a wide spectrum of organizations, varying in size, scope, and focus areas, yet unified by their commitment to advancing social causes and addressing societal needs. NGOs fulfill many roles that complement and sometimes challenge government actions and policies. One of their primary roles is advocacy, where NGOs champion causes related to human rights, environmental sustainability, gender equality, and social justice^[11]^. NGOs pressure policymakers through lobbying, campaigning, and public awareness initiatives to enact legislation, policies, and practices that promote positive social change and protect vulnerable populations. In addition to advocacy, NGOs are crucial in service delivery, particularly in sectors where governmental resources are limited or inaccessible. For example, NGOs provide essential services such as primary healthcare, vaccination campaigns, maternal and child health programs, and disease prevention and control initiatives. They often operate in remote or underserved areas where government infrastructure is inadequate, effectively bridging gaps in healthcare provision and improving health outcomes for marginalized communities^[12]^. Education is another area where NGOs significantly contribute by promoting literacy, vocational training, and access to quality education for children and adults alike. NGOs establish schools, offer scholarships, develop educational resources, and implement teacher training programs to enhance educational opportunities in underserved regions. By investing in education, NGOs empower individuals and communities to break the cycle of poverty and achieve sustainable development. Furthermore, NGOs are pivotal in humanitarian response efforts during emergencies and crises, providing life-saving assistance such as food aid, shelter, clean water, and medical care to displaced and disaster-affected communities^[13]^. Their agility and ability to mobilize resources enable NGOs to respond swiftly to humanitarian emergencies, mitigating the immediate impact of crises and supporting long-term recovery and resilience-building efforts. NGOs also contribute to economic development through microfinance initiatives, entrepreneurship programs, and sustainable livelihood projects that empower individuals to generate income, create employment opportunities, and foster economic growth at the grassroots level. NGOs promote economic inclusion and capacity-building and contribute to poverty alleviation and SDGs^[14]^. The impact of NGOs is profound and far-reaching, influencing social, political, and economic landscapes globally. NGOs have been instrumental in combating infectious diseases, promoting reproductive health, and advancing universal health coverage agendas in healthcare. For instance, organizations like Médecins Sans Frontières (Doctors Without Borders) provide emergency medical care in conflict zones and humanitarian crises, saving countless lives and advocating for improved healthcare access worldwide^[15]^. In education, NGOs enhance educational quality and accessibility, particularly for marginalized and vulnerable populations such as girls, refugees, and children from low-income families. Initiatives like the Global Partnership for Education and Room to Read focus on expanding access to education, improving learning outcomes, and promoting educational equity globally^[16]^. NGOs also play a crucial role in promoting environmental sustainability and combating climate change through conservation projects, sustainable development initiatives, and advocacy for environmental protection policies. Organizations such as Greenpeace and WWF (World Wide Fund for Nature) advocate for biodiversity conservation, climate action, and sustainable resource management to mitigate environmental degradation and promote a more sustainable future. Furthermore, NGOs contribute to peacebuilding and conflict resolution efforts by fostering dialogue, promoting reconciliation, and addressing root causes of conflict through community-based initiatives^[17]^. Organizations like the International Crisis Group and Search for Common Ground work to prevent and resolve conflicts, promote peacebuilding, and support post-conflict reconstruction and reconciliation processes^[18]^. Regarding governance and accountability, NGOs play a crucial role in promoting transparency, accountability, and good governance practices within their organizations and in broader societal contexts. They engage in monitoring and advocacy efforts to hold governments and corporations accountable for their actions, promote democratic participation, and safeguard human rights. Despite their significant contributions, NGOs face many challenges that impact their effectiveness and sustainability^[19]^. One major challenge is funding instability, as NGOs often rely on donor contributions, grants, and fundraising efforts that fluctuate due to economic downturns, shifting donor priorities, or political factors. Securing sustainable funding sources and diversity is crucial for NGOs to maintain operational continuity and expand their impact over the long term. Another critical challenge is regulatory constraints and bureaucratic hurdles governments impose, which can restrict NGO activities, limit their autonomy, or hinder their ability to operate effectively^[20]^. In some countries, restrictive legislation, registration requirements, and censorship laws may impede NGOs’ ability to advocate freely, deliver services, or collaborate with international partners. Moreover, NGOs often face logistical challenges, especially when operating in conflict zones, remote areas, or regions with inadequate infrastructure. Limited access to transportation, communication networks, and basic amenities can hamper humanitarian relief efforts and delay the delivery of essential services to vulnerable populations. In addition, maintaining organizational integrity and upholding ethical standards are paramount concerns for NGOs^[21]^. Governance transparency, stakeholder accountability, and ethical fundraising practices are critical to maintaining public trust and credibility. NGOs must adhere to rigorous standards of conduct, financial transparency, and compliance with legal and regulatory requirements to safeguard their reputation and ensure sustainable operations^[22]^. Furthermore, navigating complex geopolitical dynamics and negotiating partnerships with diverse stakeholders, including governments, international organizations, corporations, and local communities, requires diplomacy, strategic collaboration, and cultural sensitivity. Effective partnerships are essential for maximizing resources, leveraging expertise, and achieving collective impact in addressing complex global challenges^[23]^.

PPPs are another type of funding, where public and private entities share responsibilities, risks, and rewards to address HIV/AIDS challenges. In infrastructure development, for example, PPPs facilitate the construction, maintenance, and operation of public infrastructure projects such as transportation systems, energy facilities, and utilities^[24]^. Private sector partners contribute capital investment, technical expertise, and operational efficiency, while governments provide regulatory oversight, land acquisition, and long-term planning to ensure public interest and accountability. PPPs aim to improve healthcare access, quality, and efficiency through joint initiatives that enhance healthcare infrastructure, expand service delivery capabilities, and innovate healthcare delivery models^[25]^. Private-sector healthcare providers bring clinical expertise, technology innovation, and management efficiency, complementing public-sector investments in healthcare facilities, workforce training, and patient care services. PPPs in healthcare often focus on improving healthcare outcomes, reducing healthcare disparities, and promoting universal health coverage through collaborative governance frameworks and strategic partnerships^[26]^. Education reform initiatives leveraging PPPs seek to enhance educational quality, accessibility, and equity by mobilizing private sector resources, expertise, and innovation to support educational institutions, develop curriculum frameworks, and implement educational technology solutions^[27]^. Private sector partners contribute funding for school infrastructure development, teacher training programs, and educational material procurement. At the same time, governments provide policy guidance, curriculum standards, and regulatory oversight to ensure academic integrity and accountability^[28]^. Environmental sustainability initiatives supported by PPPs aim to address environmental challenges such as climate change mitigation, natural resource conservation, and sustainable development through collaborative efforts that integrate private sector environmental expertise, technology innovation, and sustainable business practices with government policies, regulatory frameworks, and community engagement strategies^[29]^. PPPs in environmental sustainability focus on promoting renewable energy adoption, improving waste management systems, protecting biodiversity, and enhancing environmental resilience through cross-sector collaboration and shared environmental stewardship responsibilities^[30]^. Economic revitalization efforts facilitated by PPPs aim to stimulate economic growth, create job opportunities, and foster entrepreneurship through joint initiatives that leverage private sector investments in infrastructure development, small business support programs, and economic development projects. Private sector partners bring financial resources, business acumen, and market access opportunities. At the same time, governments provide policy incentives, regulatory reforms, and infrastructure investments to attract private sector investments, stimulate local economies, and promote sustainable economic development^[31]^. Despite their potential benefits, PPPs face regulatory complexities, contractual disputes, financial risks, and governance issues that require careful planning, transparency, and stakeholder engagement to mitigate risks and ensure project success. Effective PPPs require clear objectives, robust legal frameworks, and performance-based contracts that align incentives, define roles and responsibilities, and promote accountability among public and private sector partners^[32]^.

While various types of funding, including government, private sector, NGOs, and PPPs, play essential roles in addressing HIV/AIDS, the sustainability of these efforts is increasingly challenged by economic factors. The global economic environment significantly affects the financial capacities of donor countries, international organizations, and philanthropic entities, making sustained funding for HIV/AIDS programs more difficult to secure.^[33]^. Economic downturns, recessions, and financial crises can reduce foreign aid budgets as donor countries prioritize domestic economic stabilization over international health assistance. For instance, during the global financial crisis of 2008-2009, several high-income countries reduced their foreign aid contributions, which had direct repercussions on funding for HIV/AIDS programs worldwide^[34]^. Similarly, the economic impacts of the COVID-19 pandemic have strained national budgets and redirected resources toward immediate pandemic response efforts, potentially jeopardizing the continuity of HIV/AIDS funding commitments. Exchange rate fluctuations also impact HIV/AIDS funding by affecting the value of financial commitments made in foreign currencies. When donor currencies depreciate relative to the currencies of recipient countries, the actual amount of funding received can decrease, leading to shortfalls in program budgets^[35]^. This is particularly problematic for countries that rely heavily on external funding to support their HIV/AIDS initiatives. For example, a decline in the value of the US dollar against local currencies in African countries can reduce the purchasing power of US-funded HIV/AIDS programs, necessitating adjustments in program scale or scope to align with available resources^[36]^. Local economic conditions within recipient countries also play a crucial role in determining the availability and effectiveness of HIV/AIDS funding. In low- and middle-income countries, economic instability, inflation, and budget deficits can constrain government spending on health, including HIV/AIDS programs. Governments facing economic challenges may struggle to allocate sufficient domestic resources for HIV/AIDS prevention, treatment, and care, increasing their dependence on external funding^[37]^. Furthermore, economic hardships can exacerbate poverty and unemployment, leading to higher HIV vulnerability and transmission rates and placing additional strain on already limited healthcare resources. Economic inequalities within countries can also affect the distribution and impact of HIV/AIDS funding^[38]^. In regions with significant income disparities, marginalized and economically disadvantaged populations may have limited access to HIV prevention and treatment services despite the availability of funding. Addressing these inequities requires targeted interventions and inclusive funding strategies that ensure resources reach those most in need. For instance, targeted funding initiatives aimed at supporting community-based organizations working with vulnerable groups such as sex workers, PWID, and MSM can help bridge gaps in service provision and reduce HIV transmission rates among high-risk populations^[39]^. The private sector’s economic involvement in HIV/AIDS funding presents both opportunities and challenges. On the one hand, private sector engagement through corporate social responsibility initiatives, philanthropic donations, and PPPs can provide additional financial resources for HIV/AIDS programs. Companies in industries such as pharmaceuticals, healthcare, and finance have contributed significantly to HIV/AIDS funding, supporting research, treatment access, and public health campaigns. For example, pharmaceutical companies have developed and distributed antiretroviral drugs at reduced costs or through donation programs, enhancing treatment accessibility for PLWHA in low-income countries^[40]^. On the other hand, economic pressures on the private sector, such as profit maximization, market competition, and shareholder expectations, can limit the extent and sustainability of private sector contributions to HIV/AIDS funding. Companies facing economic downturns or financial challenges may scale back their funding commitments or withdraw from partnerships, creating uncertainties in funding continuity^[41]^. Additionally, the commercial interests of pharmaceutical companies may influence drug pricing and accessibility, posing challenges for governments and NGOs working to provide affordable HIV/AIDS treatment in resource-limited settings. Innovation in funding mechanisms is essential to address economic challenges and ensure sustainable HIV/AIDS funding. Innovative financing models, such as social impact bonds, development impact bonds, and blended finance, combine public and private sector resources to leverage additional funding for HIV/AIDS programs^[42]^. These models align financial returns with health outcomes, incentivizing investment in effective interventions and fostering collaboration between diverse stakeholders. For example, social impact bonds have been used to fund HIV prevention programs, with private investors providing upfront capital and receiving returns based on the achievement of predefined health targets, such as reductions in new HIV infections^[43]^. Moreover, economic considerations are integral to the sustainability of HIV/AIDS funding at the local level. Strengthening domestic health financing systems and enhancing economic resilience are crucial for reducing dependency on external funding and ensuring long-term program sustainability. Governments can explore strategies such as increasing health budget allocations, implementing health insurance schemes, and leveraging domestic revenue sources, including taxes and social health contributions, to support HIV/AIDS programs. For instance, some countries have introduced “sin taxes” on tobacco, alcohol, and sugary beverages, with a portion of the revenue earmarked for health initiatives, including HIV/AIDS programs^[44]^. International financial institutions, such as the World Bank and the International Monetary Fund (IMF), are also significant in shaping the economic landscape for HIV/AIDS funding. These institutions provide financial support, technical assistance, and policy guidance to countries grappling with economic challenges. Through initiatives such as the Global Fund to Fight AIDS, Tuberculosis, and Malaria, the World Bank and other multilateral organizations mobilize resources and coordinate efforts to address HIV/AIDS in low- and middle-income countries^[45]^. However, the conditionalities attached to financial assistance, such as structural adjustment programs and fiscal austerity measures, can sometimes limit governments’ flexibility in allocating resources for health, necessitating careful consideration of the broader economic impacts of such policies on HIV/AIDS funding^[46]^.

Political stability is another key challenge, as it is a foundational determinant of effective HIV/AIDS funding and program implementation. Stable political environments provide the conditions for sustained government commitment, consistent policy implementation, and reliable funding flows. In politically stable countries, governments are more likely to allocate resources to health sectors, including HIV/AIDS programs, and implement long-term strategies that address the epidemic comprehensively. For instance, countries with stable political systems, such as Botswana and Rwanda, have demonstrated significant progress in reducing HIV prevalence and improving access to ART through sustained government investment and robust policy frameworks^[1,2]^. In contrast, countries experiencing political instability, such as conflict or frequent changes in government, often struggle to maintain consistent HIV/AIDS funding and program implementation. Political instability can disrupt health services, hinder ART delivery, and compromise the effectiveness of prevention and treatment efforts. In conflict-affected regions like South Sudan and the Democratic Republic of Congo, the breakdown of health systems due to political instability has severely impacted HIV/AIDS interventions, leading to increased transmission rates and reduced access to care^[3]^. Government commitment to HIV/AIDS funding and policy implementation is a critical factor influenced by political will and leadership. Political leaders who prioritize HIV/AIDS as a public health issue are more likely to mobilize resources, enact supportive policies, and champion initiatives that address the epidemic. High-level political commitment can galvanize national and international support, attract donor funding, and foster partnerships with civil society organizations. For example, in Uganda, strong political leadership under President Yoweri Museveni in the early 1990s was instrumental in the country’s initial success in reducing HIV prevalence through comprehensive prevention and treatment programs^[4]^. Conversely, a lack of political commitment can undermine HIV/AIDS funding and hinder progress. In some countries, HIV/AIDS may not be prioritized due to competing political agendas, stigmatization of affected populations, or denial of the epidemic’s severity. This lack of prioritization can result in inadequate funding, weak policy implementation, and insufficient support for affected communities. For instance, in Russia, political reluctance to acknowledge and address the HIV/AIDS epidemic among key populations, such as PWID, has limited the effectiveness of harm reduction programs and contributed to rising HIV incidence rates^[5]^. Policy frameworks and legal environments play a crucial role in shaping HIV/AIDS funding and program implementation. Comprehensive and inclusive policies that promote human rights, gender equality, and access to healthcare are essential for effective HIV/AIDS interventions. National HIV/AIDS strategies that align with international guidelines, such as the UNAIDS 95-95-95 targets, provide a roadmap for coordinated and targeted responses. Countries with well-developed policy frameworks, such as South Africa and Thailand, have made significant strides in scaling up ART coverage, reducing new infections, and improving health outcomes for people living with HIV^[6,7]^. However, restrictive policies and legal barriers can impede HIV/AIDS funding and undermine program effectiveness. Criminalization of key populations, such as sex workers, MSM, and PWID, creates barriers to accessing HIV prevention and treatment services. Discriminatory laws and policies exacerbate stigma, discourage individuals from seeking care, and limit the reach of HIV/AIDS interventions. In countries like Nigeria and Uganda, punitive laws targeting LGBT communities have hindered HIV prevention efforts and restricted funding for community-based organizations working with these populations^[8,9]^. International political dynamics also significantly influence HIV/AIDS funding. Global health initiatives, such as the PEPFAR and the Global Fund to Fight AIDS, Tuberculosis, and Malaria, have been instrumental in mobilizing resources and supporting national HIV/AIDS programs. The political commitment of donor countries and international organizations to global health priorities determines the availability and sustainability of funding for HIV/AIDS. For example, PEPFAR, initiated by the United States in 2003, has provided substantial funding and technical assistance to countries heavily affected by HIV/AIDS, contributing to significant reductions in HIV-related mortality and morbidity^[10]^. However, changes in political leadership and foreign policy priorities in donor countries can impact the continuity and scale of HIV/AIDS funding. Shifts in political agendas, economic policies, and bilateral relations can lead to fluctuations in funding levels, creating uncertainties for recipient countries. For instance, changes in the US administration have occasionally resulted in policy shifts that affect PEPFAR funding allocations and priorities^[11]^. Furthermore, geopolitical considerations and diplomatic relations can influence HIV/AIDS funding and collaboration. International sanctions, trade policies, and diplomatic tensions can impact the flow of resources and the ability of countries to access essential medicines and technologies for HIV/AIDS treatment. For example, economic sanctions on countries like Iran and Venezuela have restricted their access to life-saving medications and hindered their HIV/AIDS response efforts^[12,13]^. Additionally, political pressures and strategic interests may shape the allocation of HIV/AIDS funding, with donor countries sometimes prioritizing funding to regions of geopolitical importance or strategic alliances. Political advocacy and civil society engagement are essential for influencing HIV/AIDS funding and policy decisions. Civil society organizations, including NGOs, community-based organizations, and advocacy groups, play a crucial role in raising awareness, mobilizing resources, and holding governments accountable for their commitments to HIV/AIDS. Advocacy efforts can influence political leaders to prioritize HIV/AIDS, allocate adequate funding, and implement supportive policies. For example, the Treatment Action Campaign (TAC) in South Africa successfully advocated for the government to expand access to ART and address the HIV/AIDS epidemic through legal challenges and public campaigns^[14]^. However, political repression, restrictions on civil society activities, and limited political space can hinder advocacy efforts and limit the impact of civil society engagement. In some countries, laws and policies that restrict freedom of association, assembly, and expression pose significant challenges for HIV/AIDS advocacy and civil society mobilization.

Additionally, stigma associated with HIV/AIDS remains a significant barrier to effective funding and intervention. HIV-related stigma can manifest in various forms, including social ostracism, discrimination, and internalized stigma among those living with the virus. These manifestations of stigma deter individuals from seeking testing, treatment, and support services, ultimately undermining the effectiveness of funded programs. Stigmatization leads to underreporting of HIV cases and hampers efforts to track and address the epidemic^[1]^ accurately. For instance, in many parts of sub-Saharan Africa, the pervasive stigma surrounding HIV/AIDS contributes to a significant gap between the estimated number of people living with HIV and those who have been diagnosed and linked to care^[2]^. Public awareness about HIV/AIDS is crucial for reducing stigma and encouraging community support for funding and intervention efforts. High levels of public awareness can lead to a better understanding and acceptance of HIV/AIDS as a chronic but manageable condition, fostering an environment where people are more likely to seek testing and treatment without fear of judgment or discrimination. Public awareness campaigns have been instrumental in changing perceptions and attitudes towards HIV/AIDS in various contexts. For example, widespread education efforts in Uganda during the early 2000s, including the ABC (Abstinence, Be Faithful, Condom use) campaign, significantly increased public awareness and reduced stigma, contributing to a decline in HIV prevalence^[3]^. However, public awareness alone is insufficient if it is not accompanied by accurate information and cultural sensitivity. Misinformation and myths about HIV/AIDS can perpetuate stigma and hinder efforts to promote testing and treatment. In many communities, misconceptions about HIV transmission, such as the belief that it can be spread through casual contact, persist despite public awareness campaigns. These misconceptions fuel stigma and create barriers to funding and implementing effective programs. Addressing these challenges requires continuous public education efforts that provide accurate information about HIV transmission, prevention, and treatment in culturally appropriate ways^[4]^. The intersection of stigma and public awareness with funding can be observed in the allocation of resources for HIV/AIDS programs. Stigma and lack of awareness can lead to insufficient political and financial support for HIV/AIDS interventions. When public opinion is shaped by stigma and misinformation, there is often less political will to allocate necessary resources for comprehensive HIV/AIDS programs. Politicians and policymakers may be reluctant to prioritize HIV/AIDS funding due to fear of backlash or controversy, especially in regions where HIV/AIDS is highly stigmatized. This reluctance can result in inadequate funding for prevention, treatment, and care services, exacerbating the epidemic^[5]^. Moreover, stigma and low public awareness can affect the willingness of individuals and communities to participate in HIV/AIDS research and clinical trials. Participation in research is crucial for developing new treatments, understanding the epidemic, and evaluating the effectiveness of interventions. However, fear of stigma and discrimination can deter people from enrolling in studies, particularly in communities where HIV is highly stigmatized. This reluctance can slow the progress of research and limit the availability of evidence-based interventions, ultimately affecting the allocation of funding for HIV/AIDS programs^[6]^. In addition to affecting funding and research, stigma and public awareness influence the implementation and success of HIV/AIDS programs. Programs that fail to address stigma and raise public awareness may struggle to achieve their objectives, regardless of the level of funding they receive. For example, programs to increase ART coverage must also reduce stigma and improve public awareness to ensure that individuals feel safe and supported in seeking treatment. Comprehensive approaches that integrate stigma reduction and public education with medical interventions are more likely to succeed in improving health outcomes for PLWHA^[7]^. Community-based organizations (CBOs) and NGOs play a vital role in addressing stigma and raising public awareness about HIV/AIDS. These organizations often have deep ties to their communities and can effectively engage with individuals to provide education, support, and advocacy. By working at the grassroots level, CBOs and NGOs can challenge stigma, dispel myths, and promote understanding of HIV/AIDS. For example, organizations like the Treatment Action Campaign (TAC) in South Africa have been successful in mobilizing communities, advocating for the rights of PLWHA, and raising awareness about the epidemic^[8]^. Effective HIV/AIDS funding strategies must also consider the diverse and intersecting identities of people living with and affected by HIV. Intersectional stigma, which arises from multiple stigmatized identities such as being HIV-positive, LGBTQ+, a person who injects drugs, or a sex worker, can compound the challenges of accessing care and support. Programs that address HIV/AIDS must be inclusive and responsive to the needs of these marginalized populations. This requires funding for targeted interventions that provide medical care and address these groups’ unique social and structural barriers. For instance, harm reduction programs for PWID and safe spaces for LGBTQ+ individuals are critical components of comprehensive HIV/AIDS strategies^[9]^. Addressing stigma and raising public awareness is also essential for sustaining long-term funding commitments from international donors. Donor agencies and philanthropic organizations are more likely to support countries and programs that demonstrate effective use of funds, strong community engagement, and measurable outcomes. By investing in stigma reduction and public education, countries can create an enabling environment that attracts and retains donor support. For example, countries that have successfully reduced HIV stigma and increased public awareness, such as Brazil, have been able to secure sustained international funding and technical assistance for their HIV/AIDS programs^[10]^. Innovative approaches to funding that prioritize stigma reduction and public awareness can also enhance the effectiveness of HIV/AIDS interventions. Social marketing campaigns, community-led initiatives, and peer education programs are examples of innovative strategies that can be supported through targeted funding. These approaches leverage the power of social networks and community engagement to change perceptions and behaviors related to HIV/AIDS. For instance, social marketing campaigns that use popular media and influencers to promote HIV testing and treatment have been successful in reaching young people and other high-risk groups^[11]^.

### Innovative approaches to funding

Innovative funding models are essential for addressing the financial challenges of HIV/AIDS prevention, treatment, and research. One prominent innovative funding model is PPPs, which leverage the strengths and resources of both the public and private sectors to achieve common health goals. PPPs can mobilize significant financial resources and expertise that may not be available through traditional funding channels alone. For example, the partnership between the Global Fund to Fight AIDS, Tuberculosis, and Malaria and private companies has successfully raised substantial funds and improved access to treatment and prevention services in many low- and middle-income countries. These partnerships often involve the private sector providing financial contributions, in-kind donations, or technical expertise, while the public sector offers policy support, regulatory frameworks, and program implementation capabilities. By combining resources and expertise, PPPs can enhance the efficiency and impact of HIV/AIDS interventions^[1]^. Another innovative funding model uses social impact bonds (SIBs), also known as pay-for-success bonds. SIBs involve private investors providing upfront capital to fund social programs, with the government or another entity repaying the investors based on the achievement of predefined outcomes. This model shifts the financial risk from the public sector to private investors, who are incentivized to ensure the success of the funded programs. In the context of HIV/AIDS, SIBs could be used to finance prevention initiatives, such as needle exchange programs or PrEP distribution, with repayment contingent on reductions in new HIV infections. This outcome-based approach encourages greater accountability and effectiveness in program implementation, as investors are vested in achieving positive health outcomes^[2]^. Crowdfunding has emerged as another innovative funding model with the potential to support HIV/AIDS programs. Platforms like GoFundMe, Kickstarter, and Indiegogo enable individuals and organizations to raise funds directly from the public for specific projects or initiatives. Crowdfunding campaigns can be particularly effective in generating support for innovative research, community-based prevention programs, and emergency response efforts. For example, crowdfunding has been used to fund the development of new diagnostic tools, support grassroots advocacy efforts, and provide immediate assistance to individuals affected by HIV/AIDS. The decentralized nature of crowdfunding allows for a broad base of small donors to contribute, democratizing the funding process and fostering a sense of community involvement and ownership^[3]^. Corporate social responsibility (CSR) initiatives also represent an important source of innovative funding for HIV/AIDS programs. Many corporations recognize the importance of contributing to global health efforts and have established CSR programs that allocate funds and resources to address public health issues, including HIV/AIDS. For instance, pharmaceutical companies may provide antiretroviral drugs at reduced prices or donate medications to support treatment programs in low-income countries. Other corporations may fund prevention campaigns, support research initiatives, or implement workplace programs to promote HIV awareness and testing among employees. CSR initiatives provide valuable financial and in-kind support, enhance corporate reputation, and foster positive stakeholder relationships^[4]^. Innovative taxation mechanisms have been proposed as another means of generating sustainable funding for HIV/AIDS programs. One example is the “Robin Hood tax,” a small levy on financial transactions, such as stock trades and currency exchanges, that could generate significant revenue for global health initiatives. Similarly, taxes on products with negative health impacts, such as tobacco, alcohol, and sugary beverages, could be earmarked for HIV/AIDS programs. These “sin taxes” raise funds, promote healthier behaviors, and reduce the burden of related diseases. Implementing such taxes requires political will and careful design to ensure they are equitable and do not disproportionately affect low-income populations. However, when effectively implemented, innovative taxation can provide a reliable and sustainable source of funding for HIV/AIDS efforts^[5]^. Philanthropic foundations have long played a crucial role in funding HIV/AIDS programs, and some have adopted innovative approaches to maximize their impact. For example, the Bill & Melinda Gates Foundation has pioneered catalytic philanthropy, which involves making strategic investments that leverage additional funding from other sources. This approach includes providing seed funding for innovative projects, supporting the development of new technologies, and advocating for policy changes to create an enabling environment for HIV/AIDS interventions. By using their resources to catalyze broader systemic changes, philanthropic foundations can amplify their impact and drive progress in the fight against HIV/AIDS^[6]^. Conditional cash transfers (CCTs) have also shown promise as an innovative funding model for HIV/AIDS prevention. CCTs provide financial incentives to individuals or households to engage in health-promoting behaviors, such as attending HIV testing and counseling sessions, adhering to ART, or participating in prevention programs. These incentives can help overcome barriers to access and encourage positive health behaviors. For instance, a study in rural Malawi found that providing small cash payments to individuals who tested negative for sexually transmitted infections significantly reduced risky sexual behaviors and HIV incidence. CCTs can be tailored to target specific populations and behaviors, making them a flexible and effective tool for HIV/AIDS prevention^[7]^. Impact investing is another innovative funding model that has gained traction in the global health sector. Impact investors seek to generate social and financial returns by investing in businesses and projects addressing pressing social issues, including HIV/AIDS. For example, impact investors may fund companies developing new HIV diagnostic technologies, support social enterprises providing healthcare services in underserved areas, or invest in initiatives that improve access to PrEP and other preventive measures. By aligning financial incentives with social outcomes, impact investing can attract capital to high-impact HIV/AIDS programs and foster sustainable business models that improve long-term health^[8]^. Blended finance is an approach that combines public and private funding to achieve development goals, including HIV/AIDS prevention and treatment. This model leverages public funds to de-risk investments and attract private capital, increasing the resources available for HIV/AIDS programs. For example, public funds can provide guarantees or first-loss capital, reducing the financial risk for private investors and encouraging them to invest in health initiatives. Blended finance can mobilize additional resources and promote innovative solutions to complex health challenges. By aligning the interests of public and private stakeholders, this model can enhance the efficiency and impact of HIV/AIDS interventions^[9]^. Finally, innovative insurance schemes have the potential to support HIV/AIDS programs by providing financial protection and reducing the economic burden of the disease. Health insurance schemes that include coverage for HIV-related services can improve access to testing, treatment, and preventive measures. Microinsurance products, designed for low-income populations, can offer affordable coverage for essential health services, including those related to HIV/AIDS. Insurance-linked financial products, such as catastrophe bonds, can provide rapid funding for emergency response efforts during HIV outbreaks or other health crises. By mitigating financial risks and improving access to care, innovative insurance models can contribute to more resilient and sustainable HIV/AIDS programs^[10]^.

One of the most impactful technological innovations in funding is crowdfunding, which has revolutionized the way financial support is raised for HIV/AIDS initiatives. This is part of a wider movement toward innovative approaches to funding, where new strategies and technologies are being explored to enhance resource mobilization and ensure the sustainability of HIV/AIDS programs. Crowdfunding platforms like Kickstarter, GoFundMe, and Indiegogo have democratized raising funds by allowing individuals and organizations to solicit small contributions from many people, typically via the Internet. This model has effectively supported creative projects, startups, and emergency relief efforts. For instance, during the COVID-19 pandemic, crowdfunding platforms facilitated rapid fundraising for medical supplies, support for affected individuals, and research initiatives. The success of crowdfunding lies in its ability to harness the collective power of individuals who share a common interest or concern, thereby bypassing traditional funding barriers^[1]^. Another significant advancement is the use of blockchain technology in funding. Blockchain offers a decentralized, transparent, and secure way of managing financial transactions, which can enhance trust and accountability in funding processes. Cryptocurrencies, a product of blockchain technology, have emerged as an alternative funding source. Platforms like Bitcoin and Ethereum have been used to raise funds for various causes, from disaster relief to social impact projects. One notable example is the UNICEF Cryptocurrency Fund, which accepts and disburses donations in cryptocurrency to fund open-source technology benefiting children globally. Blockchain’s transparency ensures donors can track how their funds are used, increasing confidence in the funding process^[2]^. Artificial intelligence (AI) and machine learning are also transforming funding mechanisms. These technologies can analyze vast amounts of data to identify funding trends, predict successful projects, and optimize resource allocation. For example, AI-driven platforms like Donorbox and CauseVox use algorithms to recommend fundraising strategies based on historical data and donor behavior. These insights enable organizations to tailor their campaigns more effectively, increasing the likelihood of success. Additionally, AI can streamline grant application processes by automating eligibility checks and scoring applications, reducing administrative burdens and speeding up decision-making^[3]^. Peer-to-peer (P2P) lending platforms represent another technological innovation in funding. P2P lending connects borrowers directly with lenders through online platforms, bypassing traditional financial intermediaries like banks. This model has particularly benefited small businesses and individuals who might otherwise face difficulties accessing credit. Platforms like LendingClub and Prosper facilitate these transactions, providing a more accessible and often affordable funding source. The P2P model’s efficiency and lower overhead costs than traditional banking make it an attractive option for many borrowers^[4]^. Fintech solutions, which combine finance and technology, have also revolutionized funding for social and environmental causes. Mobile banking and digital payment systems, such as M-Pesa in Kenya, have significantly improved financial inclusion, enabling millions of unbanked individuals to access financial services. These technologies facilitate microfinance and microloans, empowering small entrepreneurs and communities to invest in their businesses and improve their livelihoods. The convenience and accessibility of mobile financial services have transformed how people save, borrow, and spend money, driving economic development at the grassroots level^[5]^. Another area where technological innovations have made a significant impact is in the realm of impact investing. Impact investing is investments made to generate positive social or environmental impact alongside financial returns. Technology has enabled the development of sophisticated platforms that connect investors with impact-driven projects. For example, platforms like ImpactUs and Swell Investing provide tools for investors to track their investments’ social and environmental performance. Data analytics and reporting technologies ensure transparency and accountability, allowing investors to make informed decisions that align with their values^[6]^. Digital wallets and payment gateways have facilitated more efficient and secure funding mechanisms. These technologies allow for seamless transactions across borders, reducing the barriers to international funding. Platforms like PayPal, Stripe, and Square enable individuals and organizations to receive donations and payments from anywhere in the world, fostering global collaboration and support. Integrating digital wallets with fundraising platforms has streamlined the donation process, making it easier for donors and recipients to access funds^[7]^. The integration of social media with fundraising has also been a game-changer. Social media platforms like Facebook, Twitter, and Instagram have become powerful tools for fundraising campaigns, enabling organizations to reach a vast audience quickly and engage them in their cause. Features like Facebook Fundraisers and Instagram’s donation stickers allow users to contribute directly through social media, making the donation process more accessible and immediate. The viral nature of social media can amplify fundraising efforts, as campaigns can be shared widely, reaching potential donors who might not have been accessible through traditional channels^[8]^. Geospatial technologies and data analytics have enhanced the ability to target funding where it is most needed. Geographic Information Systems (GIS) and other spatial analysis tools can map needed areas and track the impact of funded projects. For example, these technologies can help identify the most affected areas and allocate resources more effectively during natural disasters. By visualizing data geographically, funders can make more informed decisions about where to direct their resources, ensuring that aid reaches the most vulnerable populations^[9]^. One of the challenges of technological innovations in funding is ensuring equitable access to these technologies. While digital platforms have somewhat democratized funding, a digital divide remains, particularly in low-income and rural areas. Access to the Internet and digital literacy are prerequisites for benefiting from these innovations. Therefore, efforts to expand internet access and provide digital education are crucial for maximizing the impact of technological advancements in funding. Initiatives like Google’s Project Loon, which aims to provide internet access to remote areas using high-altitude balloons, represent steps toward bridging this gap^[10]^. Another challenge is maintaining security and privacy in digital funding platforms. As these platforms handle sensitive financial information, they are potential cyberattack targets. Ensuring robust cybersecurity measures is essential to protect users’ data and maintain trust in the system. With its decentralized and encrypted nature, blockchain technology offers potential solutions for enhancing security in digital transactions. However, ongoing vigilance and innovation in cybersecurity are necessary to stay ahead of emerging threats^[11]^. Regulatory and legal frameworks also need to evolve to keep pace with technological innovations in funding. Governments and regulatory bodies must balance fostering innovation and protecting consumers. For instance, the regulation of cryptocurrencies varies widely across countries, creating uncertainty for users and investors. Clear and consistent regulations can provide a stable environment for technological advancements in funding to thrive while ensuring consumer protection and financial stability^[12]^. Looking to the future, several trends and emerging technologies hold promise for further transforming funding mechanisms. One such trend is the rise of decentralized finance (DeFi). DeFi leverages blockchain technology to create decentralized financial systems without traditional intermediaries. This can reduce costs and increase access to financial services, particularly in underserved regions. DeFi platforms like Compound and Aave enable users to lend and borrow assets directly, providing new opportunities for funding and investment^[13]^. Other emerging technologies are artificial intelligence and big data analytics, which enhance transparency and efficiency in funding. AI can analyze large datasets to identify patterns and trends, optimize resource allocation, and predict the impact of funded projects. This data-driven approach can improve decision-making and ensure that funds are directed to where they can achieve the greatest impact. For example, AI-powered platforms can assess the effectiveness of different interventions in public health, enabling funders to allocate resources more strategically^[14]^. The development of smart contracts on blockchain platforms represents another significant innovation. Smart contracts are self-executing contracts with the terms of the agreement directly written into code. They automatically execute transactions when predefined conditions are met, reducing the need for intermediaries and increasing efficiency. In the funding context, smart contracts can ensure that funds are released only when specific milestones are achieved, enhancing accountability and reducing the risk of mismanagement. For instance, a smart contract could only release funds for a construction project when verifiable progress is made^[15]^. Tokenization is another promising development in the realm of funding. Tokenization involves converting rights to an asset into a digital token on a blockchain. This can create new opportunities for fractional ownership and investment in previously inaccessible assets to many investors. For example, real estate tokenization allows investors to purchase fractions of a property, making it easier to diversify investments and raise capital. This technology can open up new funding avenues for projects that require large capital investments^[16]^.

The Global Fund to Fight AIDS, Tuberculosis, and Malaria stands as a prominent example of a successful funding model. Such case studies illustrate the effectiveness of collaborative, large-scale funding efforts and offer valuable lessons for shaping future strategies to combat HIV/AIDS. Established in 2002, the Global Fund is a partnership between governments, civil society, the private sector, and people affected by diseases. Its funding model is based on multi-year pledges from donor countries, complemented by contributions from the private sector and foundations. This model emphasizes performance-based funding, where grants are disbursed based on the achievement of predefined results. The Global Fund has mobilized and invested over $50 billion, significantly contributing to reducing mortality rates from these diseases. Its success lies in its ability to pool resources from various donors and allocate them efficiently to high-impact interventions^[1]^.

Another successful case study is the Gavi, the Vaccine Alliance. Gavi was created in 2000 to improve access to vaccines in low-income countries. Its innovative funding model includes direct contributions from governments, private sector partners, and innovative financing mechanisms like the International Finance Facility for Immunization (IFFIm). IFFIm raises funds by issuing bonds in the capital markets, backed by long-term donor pledges. This approach allows Gavi to access funding upfront and ensure a stable supply of vaccines. As a result, Gavi has helped immunize over 888 million children and prevent more than 15 million future deaths. The strategic use of market-based instruments alongside traditional funding has been a cornerstone of Gavi’s success^[2]^. The Green Climate Fund (GCF) represents another innovative and successful funding model. Established in 2010 under the United Nations Framework Convention on Climate Change (UNFCCC), the GCF aims to support developing countries in combating climate change. The GCF’s funding model is based on contributions from developed countries, which are then allocated to climate projects through a rigorous selection process. The GCF also leverages private sector investment through its Private Sector Facility, which aims to mobilize private capital for climate action. The Fund has approved over $8 billion in projects, leveraging additional co-financing from the private sector and other sources. The GCF’s ability to blend public and private finance has been critical in scaling climate investments^[3]^. In the realm of education, the Global Partnership for Education (GPE) provides a compelling case study. GPE was founded in 2002 to strengthen education systems in the world’s poorest countries. Its funding model relies on contributions from donor countries, multilateral organizations, and private foundations. GPE employs a results-based financing approach, where funds are disbursed based on the achievement of specific education outcomes. This model incentivizes countries to improve education quality and access. GPE has allocated over $7 billion to support education in 70 countries, significantly improving enrollment and learning outcomes. The partnership’s emphasis on country ownership and results has been key to its effectiveness^[4]^.

The Bill & Melinda Gates Foundation provides an exemplary model of philanthropic funding. Established in 2000, the foundation has committed over $50 billion to various global health, development, and education initiatives. Its funding model is characterized by large-scale, strategic investments in high-impact areas, often collaborating with other donors and organizations. The foundation’s approach includes rigorous evaluation and a focus on scalability and sustainability. For instance, in partnership with the World Health Organization, UNICEF, and Rotary International, its funding for polio eradication has contributed to a 99% reduction in polio cases worldwide. The Gates Foundation’s strategic use of grants and its emphasis on partnerships have been instrumental in its success^[5]^. The case of microfinance institutions (MFIs) like Grameen Bank in Bangladesh demonstrates a successful funding model for poverty alleviation. Founded by Muhammad Yunus in 1983, Grameen Bank provides small loans to people experiencing poverty, particularly women, to start or expand small businesses. The bank’s funding model relies on donor funds, loans from international development organizations, and reinvested profits. Borrowers form small groups to guarantee each other’s loans, fostering a sense of community and accountability. Grameen Bank’s model has been replicated globally, lifting millions out of poverty through access to financial services. The success of MFIs like Grameen Bank underscores the potential of microfinance as a sustainable funding model for development^[6]^. The venture capital (VC) funding model has successfully driven innovation in the technology sector. Venture capital firms fund startups and early-stage companies with high growth potential in exchange for equity. This model has fueled the growth of numerous technology giants, such as Google, Facebook, and Amazon. Venture capital funding is characterized by high risk and high reward, with VCs often providing not only capital but also strategic guidance and mentorship. The success of the VC model lies in its ability to identify and nurture innovative ideas, contributing to the rapid advancement of technology and entrepreneurship^[7]^.

Social impact bonds (SIBs) represent another innovative funding model that successfully addresses social issues. SIBs are a form of performance-based contracting where private investors provide upfront capital for social programs, and the government repays the investors with a return if the programs achieve predefined outcomes. One notable example is the Peterborough SIB in the UK, launched in 2010 to reduce reoffending rates among short-sentenced prisoners. The program reduced reoffending by 9%, leading to a positive return for investors. The SIB model aligns the interests of investors, service providers, and governments, fostering innovation and accountability in social service delivery^[8]^. The case of the Global Innovation Fund (GIF) highlights the role of flexible funding models in supporting innovation. GIF is a nonprofit organization that invests in social innovations with the potential to improve people’s lives in developing countries. Its funding model includes grants, equity, and debt investments, allowing it to support a diverse range of projects at different stages of development. A rigorous evaluation process guides GIF’s investments to identify high-impact opportunities. Since its inception in 2014, GIF has supported numerous innovations, from mobile health applications to agricultural technologies, demonstrating the effectiveness of a flexible, impact-driven funding approach^[9]^. In the renewable energy sector, the case of Germany’s Energiewende (energy transition) provides a successful model of public policy-driven funding. The German government has implemented various policies and funding mechanisms to support the transition to renewable energy, including feed-in tariffs, subsidies, and grants for research and development. These policies have attracted significant private investment and led to a substantial increase in renewable energy capacity. By 2020, renewable sources accounted for nearly 50% of Germany’s electricity consumption. The Energiewende model illustrates the power of government-led funding initiatives to drive large-scale transitions to sustainable energy^[10]^. The example of the Wellcome Trust, a global charitable foundation based in the UK, highlights the impact of endowment-based funding models. The Wellcome Trust manages an endowment valued at over £29 billion, generating income to fund various health and research initiatives. This funding model provides a stable and sustainable funding source, enabling long-term planning and investment in high-risk, high-reward research. The Wellcome Trust’s support for groundbreaking medical research, including developing vaccines and treatments for infectious diseases, has profoundly impacted global health. The success of the endowment-based model lies in its ability to provide consistent funding independent of economic cycles^[11]^.

Another successful funding model is represented by corporate social responsibility (CSR) initiatives. Many corporations allocate a portion of their profits to CSR programs that address social and environmental issues. One notable example is the Tata Group in India, which has a long philanthropy and social responsibility history. The Tata Group funds various education, healthcare, and rural development initiatives through its trusts and foundations. The group’s commitment to CSR has contributed to social development and enhanced its corporate reputation and stakeholder relations. The Tata Group’s integrated approach to business and social responsibility exemplifies the potential of corporate funding models to drive positive change^[12]^. The case of the European Union’s Horizon 2020 program demonstrates the success of large-scale public funding for research and innovation. Horizon 2020, which ran from 2014 to 2020, was the EU’s largest research and innovation program, with a nearly €80 billion budget – the program funded various projects, from healthcare and climate action to digital technologies and space exploration. Based on peer review and excellence, Horizon 2020’s competitive funding model has supported groundbreaking research and innovation, contributing to Europe’s scientific and technological leadership. The program’s emphasis on collaboration and interdisciplinary research has been key to its success^[13]^. In the arts and culture sector, the case of the National Endowment for the Arts (NEA) in the United States illustrates a successful public funding model. The NEA provides grants to support artistic excellence and access to the arts across the country. Its funding model includes competitive grants, partnerships, and initiatives that leverage private and local funding. The NEA’s grants have supported thousands of artists, organizations, and projects, fostering cultural enrichment and community development. The NEA’s success lies in adapting its funding priorities to evolving cultural and societal needs while maintaining a rigorous and transparent grant-making process^[14]^. Lastly, the case of the crowdfunding campaign for the Pebble smartwatch exemplifies the power of grassroots funding models. Pebble, one of the first smartwatches, raised over $10 million on Kickstarter in 2012, becoming the most funded project on the platform at that time. A compelling product, effective use of social media, and strong community engagement drove the campaign’s success. Pebble’s

### Key stakeholders and contributions

Among the various stakeholders contributing to the fight against HIV/AIDS, international organizations play a pivotal role. A prime example is the UNAIDS, which has been instrumental in coordinating global efforts to address the epidemic. Established in 1996, UNAIDS leads and coordinates the global response to HIV/AIDS, working to achieve its vision of zero new HIV infections, zero discrimination, and zero AIDS-related deaths. UNAIDS plays a crucial role in setting global targets, such as the 90-90-90 goals aimed at diagnosing 90% of all people living with HIV, providing ART to 90% of those diagnosed, and achieving viral suppression for 90% of those treated by 2020. These targets have been extended with new goals for 2030, focusing on ending the AIDS epidemic as a public health threat^[1]^. UNAIDS also monitors and reports on the global HIV/AIDS epidemic, providing vital data and analysis that inform policy and programmatic decisions. Through its Global AIDS Monitoring framework, UNAIDS collects and disseminates data on HIV prevalence, incidence, and treatment coverage, enabling countries to track their progress and identify gaps. This data-driven approach ensures that interventions are based on the latest evidence and are targeted where they are needed most. Furthermore, UNAIDS advocates for increased political commitment and funding for HIV/AIDS programs, working with governments, civil society, and the private sector to sustain and scale up the response^[2]^. The World Health Organization (WHO) is another key international player in the HIV/AIDS response. WHO develops and disseminates technical guidelines and standards for HIV prevention, diagnosis, treatment, and care. These guidelines are based on rigorous scientific evidence and are regularly updated to reflect the latest advancements in the field. For example, WHO’s consolidated guidelines on the use of antiretroviral drugs for treating and preventing HIV infection have been instrumental in standardizing and improving ART regimens worldwide. WHO also provides technical assistance to countries, helping them to implement these guidelines and strengthen their health systems to deliver effective HIV services^[3]^. In addition to its normative and technical work, WHO plays a crucial role in promoting public health interventions that address the broader determinants of HIV vulnerability, such as sexual and reproductive health, gender inequality, and social stigma. By integrating HIV services with other health programs, such as those for tuberculosis, maternal and child health, and sexual and reproductive health, WHO promotes a holistic and integrated approach to HIV/AIDS care. This approach enhances the efficiency and effectiveness of health services, ensuring that people living with HIV receive comprehensive and person-centered care^[4]^. The Global Fund to Fight AIDS, Tuberculosis, and Malaria is one of the largest international financiers of HIV/AIDS programs. Since its inception in 2002, the Global Fund has mobilized and invested billions of dollars to support HIV prevention, treatment, and care services in low- and middle-income countries. The Global Fund operates through a unique partnership model, working with governments, civil society, the private sector, and affected communities to design and implement country-led programs. This collaborative approach ensures that interventions are tailored to each country’s specific needs and contexts and that communities are actively involved in decision-making processes^[5]^. The Global Fund’s investments have had a significant impact on the HIV/AIDS epidemic, contributing to substantial increases in ART coverage, reductions in new HIV infections, and declines in AIDS-related deaths. For instance, by the end of 2019, Global Fund-supported programs had provided HIV treatment to more than 20 million people and distributed over 1.2 billion condoms. The Global Fund also emphasizes the importance of strengthening the health system and supporting initiatives to improve laboratory services, supply chain management, and health workforce capacity. By addressing these systemic challenges, the Global Fund helps to create a more resilient and sustainable health infrastructure that can effectively respond to HIV/AIDS and other health threats^[6]^. PEPFAR, the U.S. PEPFAR, is another critical international initiative in the fight against HIV/AIDS. Launched in 2003, PEPFAR is the largest commitment by any nation to address a single disease in history. PEPFAR’s comprehensive approach includes funding for HIV prevention, treatment, care, and support services, as well as efforts to strengthen health systems and address social determinants of health. PEPFAR works in partnership with governments, NGOs, and the private sector in more than 50 countries, focusing on regions with the highest burden of HIV/AIDS^[7]^. PEPFAR’s investments have yielded impressive results, significantly expanding access to HIV services and reducing the impact of the epidemic. For example, as of September 2020, PEPFAR-supported programs had provided ART to nearly 18.2 million people and prevented millions of HIV infections through interventions such as voluntary medical male circumcision and PMTCT. PEPFAR also emphasizes the importance of transparency and accountability, using data-driven approaches to monitor progress and ensure that resources are used effectively. This focus on results and impact has made PEPFAR a model for other global health initiatives^[8]^. NGOs and community-based organizations (CBOs) are also vital players in the international response to HIV/AIDS. These organizations often operate at the grassroots level, providing essential services to marginalized and hard-to-reach populations. NGOs and CBOs play a crucial role in delivering HIV prevention and treatment services, conducting outreach and education, and advocating for the rights and needs of people living with HIV. Their close connections with affected communities enable them to address the social and structural factors that contribute to HIV vulnerability, such as stigma, discrimination, and lack of access to healthcare^[9]^. One notable example of an NGO working in the HIV/AIDS field is the International AIDS Society (IAS). The IAS is the world’s largest association of HIV professionals, bringing together scientists, clinicians, and public health experts to advance research and advocate for evidence-based policies. The IAS organizes the biennial International AIDS Conference, providing a platform for sharing the latest scientific developments and fostering stakeholder collaboration. The IAS also conducts policy advocacy and capacity-building activities to ensure that the best available evidence informs the global HIV response and includes all affected populations^[10]^. Another influential NGO is the AIDS Healthcare Foundation (AHF), which operates in more than 40 countries and provides HIV prevention, testing, and treatment services to over 1.5 million people. AHF adopts a community-based approach, delivering high-quality care to underserved and marginalized populations. The organization also engages in advocacy and policy work, campaigning for greater access to affordable HIV medications and promoting the rights of people living with HIV. AHF’s integrated and patient-centered model of care has been highly effective in improving health outcomes and reducing the impact of HIV/AIDS in the communities it serves^[11]^. International partnerships and collaborations are crucial for advancing HIV/AIDS research and innovation. Initiatives such as the HIV Vaccine Trials Network (HVTN) and the International Partnership for Microbicides (IPM) bring together researchers, funders, and industry partners to develop new preventive technologies, such as vaccines and microbicides. These collaborations facilitate sharing of knowledge and resources, accelerate the research and development process, and enhance the likelihood of successful outcomes. For instance, the HVTN conducts large-scale clinical trials to test HIV vaccine candidates. At the same time, the IPM focuses on developing and disseminating microbicides that women can use to protect themselves from HIV infection^[12]^. The role of international organizations in the HIV/AIDS response extends to addressing the social and economic determinants of the epidemic. Entities such as the World Bank and the United Nations Development Programme (UNDP) recognize that poverty, inequality, and lack of access to education and employment opportunities contribute to HIV vulnerability. These organizations implement programs aimed at reducing poverty, promoting gender equality, and improving access to education and economic opportunities. By addressing these underlying determinants, they contribute to a more comprehensive and sustainable approach to HIV/AIDS prevention and care^[13]^. For example, the World Bank has supported numerous projects integrating HIV/AIDS interventions with broader development goals, such as improving access to healthcare, education, and social services. The World Bank’s Multi-Country HIV/AIDS Program (MAP) provided funding to more than 30 countries in sub-Saharan Africa, supporting community-based initiatives that addressed the health and socio-economic impacts of HIV/AIDS. These projects demonstrated that integrating HIV/AIDS interventions with broader development efforts can enhance their effectiveness and contribute to overall social and economic development^[14]^. The UNDP also plays a significant role in addressing the socio-economic dimensions of HIV/AIDS. Through its HIV, Health, and Development Strategy, UNDP works to reduce the impact of HIV on vulnerable populations and to integrate HIV responses into broader development frameworks. UNDP’s initiatives include supporting legal and policy reforms to protect the rights of people living with HIV, promoting access to justice and social protection, and addressing the gender dimensions of the epidemic. By focusing on the social determinants of health and promoting inclusive and rights-based approaches, UNDP contributes to a more equitable and effective global HIV response^[15]^.

Governments play a crucial role in formulating national HIV/AIDS strategies that guide the country’s response to the epidemic. As key stakeholders, their contributions form the backbone of coordinated efforts, alongside the support of international organizations, private sector entities, NGOs, and community-based initiatives. These strategies typically include goals and targets for reducing new HIV infections, expanding access to treatment, and improving health outcomes for people living with HIV. For instance, the National Strategic Plan (NSP) on HIV/AIDS in South Africa sets ambitious targets for reducing new infections and scaling up ART coverage. Such plans are informed by epidemiological data, scientific evidence, and input from stakeholders, including people living with HIV, healthcare providers, and civil society organizations^[1]^. Effective HIV/AIDS policies often emphasize the importance of prevention, recognizing that preventing new infections is essential for controlling the epidemic. Governments implement a range of prevention strategies, including promoting condom use, expanding access to PrEP, and supporting harm reduction programs for PWID. For example, Brazil has implemented a comprehensive HIV prevention program that includes widespread condom distribution, needle exchange programs, and public education campaigns. These efforts have contributed to a stable HIV prevalence rate in the country, demonstrating the impact of robust prevention policies^[2]^. Testing and diagnosis are also critical components of national HIV/AIDS strategies. Early diagnosis of HIV is essential for linking individuals to care and treatment, which can improve health outcomes and reduce the transmission of the virus. Governments have implemented various approaches to expand HIV testing, including community-based, home-based, and provider-initiated testing in healthcare settings. Rwanda, for example, has successfully increased HIV testing rates through a combination of community outreach and integration of HIV testing into routine health services. Such strategies ensure that more people are aware of their HIV status and can access the necessary services^[3]^. Access to ART is a cornerstone of effective HIV/AIDS treatment policies. ART not only improves the health and longevity of people living with HIV but also reduces the risk of transmitting the virus to others. Governments have made significant strides in expanding ART coverage through policies that ensure the availability, affordability, and accessibility of medications. For instance, Botswana was one of the first African countries to provide free ART to all eligible individuals, leading to substantial improvements in health outcomes and a reduction in AIDS-related deaths. Such policies demonstrate the importance of government commitment to providing life-saving treatment to those in need^[4]^. Addressing the social determinants of health is another critical aspect of HIV/AIDS policies. Governments recognize that factors such as poverty, gender inequality, and stigma contribute to the vulnerability to HIV and impact the effectiveness of the response. Policies aimed at reducing stigma and discrimination, promoting gender equality, and improving access to education and economic opportunities are essential for creating an enabling environment for HIV prevention and care. For example, India’s National AIDS Control Programme includes initiatives to reduce stigma and discrimination against people living with HIV, protect their rights, and ensure their access to healthcare and social services. Such policies help to create a more inclusive and supportive environment for people affected by HIV/AIDS^[5]^. Funding and resource allocation are vital components of effective HIV/AIDS policies. Governments must allocate sufficient financial resources to support prevention, treatment, and care programs. This includes funding for medications, healthcare infrastructure, human resources, and community-based services. Many countries rely on a combination of domestic funding and international aid to support their HIV/AIDS programs. For example, Kenya’s HIV response is funded through a mix of government resources and support from international donors, such as the Global Fund to Fight AIDS, Tuberculosis, and Malaria. Effective resource allocation ensures that HIV/AIDS programs are sustainable and can meet the needs of affected populations^[6]^. Integration of HIV services with other health services is a strategy that many governments have adopted to improve the efficiency and effectiveness of their HIV/AIDS response. Integrating HIV services with primary healthcare, sexual and reproductive health, and tuberculosis (TB) services can enhance access to comprehensive care and address comorbidities. For instance, Ethiopia has implemented an integrated approach to HIV and TB care, recognizing the high co-infection rates and the need for coordinated treatment. Such integration ensures that individuals receive holistic care and that health systems are strengthened to respond to multiple health challenges^[7]^. Governments are also critical in supporting HIV/AIDS research and innovation. Policies that promote research and development can lead to new prevention methods, treatments, and cure strategies. For example, the United States government, through the National Institutes of Health (NIH), has been a major funder of HIV/AIDS research, supporting studies on HIV vaccines, cure research, and behavioral interventions. These research efforts have contributed to significant advancements in the understanding and management of HIV/AIDS. Supporting research and fostering innovation are essential for developing new tools and strategies to combat the epidemic^[8]^. International collaboration and partnerships are important elements of national HIV/AIDS strategies. Governments work with international organizations, donors, and other countries to share knowledge, resources, and best practices. For example, the PEPFAR supports HIV/AIDS programs in multiple countries, providing funding, technical assistance, and capacity building. Such international cooperation enhances the global response to HIV/AIDS and ensures that countries can learn from each other’s experiences and leverage collective resources. Collaborative efforts are crucial for addressing the transnational nature of the HIV epidemic and achieving global health goals^[9]^. Monitoring and evaluation are key components of effective HIV/AIDS policies. Governments must establish robust systems to track the implementation and impact of their programs, identify gaps, and make data-driven decisions. Monitoring and evaluation frameworks include surveillance systems, health information systems, and programmatic assessments. For instance, Thailand has developed a comprehensive monitoring and evaluation system that tracks HIV incidence, treatment outcomes, and program performance. This data informs policy adjustments and ensures that HIV/AIDS programs are responsive to changing needs and contexts. Effective monitoring and evaluation enhance accountability and improve the effectiveness of HIV/AIDS interventions^[10]^. Addressing legal and policy barriers is another critical aspect of HIV/AIDS strategies. Governments must ensure that their legal and policy frameworks do not hinder access to HIV services or perpetuate stigma and discrimination. This includes reforming laws that criminalize HIV transmission, same-sex relationships, sex work, and drug use. For example, South Africa’s legal reforms to decriminalize same-sex relationships and protect the rights of people living with HIV have been important steps in creating a supportive environment for HIV prevention and care. Removing legal barriers and protecting human rights are essential for ensuring that all individuals can access HIV services without fear of discrimination or persecution^[11]^. Community engagement and involvement are central to the success of HIV/AIDS policies. Governments must work with communities affected by HIV/AIDS to ensure that their voices are heard and their needs are addressed. Community-based organizations play a vital role in delivering services, conducting outreach, and advocating for the rights of people living with HIV. For instance, the Thai government’s collaboration with community organizations has been instrumental in reaching key populations, such as MSM and sex workers, with HIV prevention and treatment services. Community engagement ensures that HIV/AIDS programs are culturally appropriate, accessible, and effective^[12]^.

The private sector is another critical stakeholder in the fight against HIV/AIDS. One of the most direct ways it contributes is through funding, complementing the efforts of governments, international organizations, and NGOs in addressing the epidemic. Private companies, particularly those in the pharmaceutical and healthcare industries, have made substantial financial investments in HIV/AIDS research, treatment, and prevention programs. These investments support the development of new medications, vaccines, diagnostic tools, and care delivery to affected populations. For example, pharmaceutical companies like Gilead Sciences and ViiV Healthcare have invested heavily in the development and distribution of antiretroviral drugs, which are essential for the treatment of HIV/AIDS. Gilead’s commitment to expanding access to its medications has included initiatives to reduce prices in low- and middle-income countries and partnerships with generic manufacturers to produce affordable versions of their drugs^[1]^. The private sector is also a key player in research and development (R&D) for HIV/AIDS. Pharmaceutical and biotechnology companies conduct a significant portion of the research that leads to developing new HIV treatments and prevention methods. This includes the development of antiretroviral therapies (ART), PrEP, and potential vaccines. For instance, ViiV Healthcare, a global specialist HIV company, focuses exclusively on developing new therapies for HIV. Their innovative treatments have improved the quality of life for people living with HIV and contributed to the ongoing efforts to control the epidemic. Additionally, private sector investment in R&D has led to the development of long-acting injectable antiretroviral drugs, which can improve adherence and reduce the burden of daily medication^[2]^. Beyond R&D, the private sector contributes to the fight against HIV/AIDS by implementing workplace policies and programs. Many companies have recognized the importance of addressing HIV/AIDS within their workforce and have implemented comprehensive workplace programs to support employees living with HIV. These programs often include HIV education and awareness campaigns, voluntary counseling and testing, and access to treatment and support services. For example, Anglo American, a global mining company, has implemented a robust HIV/AIDS workplace program that includes free HIV testing, counseling, and treatment for employees and their dependents. Such initiatives support employees’ health and well-being and reduce workplace stigma and discrimination^[3]^. Private sector companies also engage in PPPs to enhance the global response to HIV/AIDS. These partnerships leverage the strengths of both sectors to achieve common goals, such as increasing access to treatment, improving healthcare infrastructure, and advancing research. One notable example is the partnership between the Bill & Melinda Gates Foundation and pharmaceutical companies to develop and distribute affordable HIV treatments in low-income countries. The Gates Foundation provides funding and strategic guidance, while pharmaceutical companies contribute their drug development and distribution expertise. Such collaborations have been instrumental in expanding access to life-saving medications and improving health outcomes in resource-limited settings^[4]^. In addition to direct financial contributions and partnerships, the private sector plays a crucial role in advocacy and awareness-raising efforts. Companies can use their platforms and influence to raise public awareness about HIV/AIDS, reduce stigma, and promote testing and treatment. For instance, major corporations like Apple and Starbucks have engaged in campaigns to support World AIDS Day and other HIV/AIDS-related initiatives. By leveraging their brand recognition and reach, these companies can amplify messages about HIV prevention and treatment, reaching a broad audience and encouraging public engagement^[5]^. Corporate social responsibility (CSR) initiatives are another avenue through which the private sector contributes to the fight against HIV/AIDS. Many companies integrate HIV/AIDS-related activities into their broader CSR strategies, recognizing that supporting public health aligns with their business goals and values. For example, Johnson & Johnson has implemented CSR initiatives focused on HIV prevention and maternal and child health, including partnerships with local organizations to deliver health services and education in high-burden areas. Such CSR initiatives demonstrate the private sector’s commitment to addressing global health challenges and contributing to the sustainability of HIV/AIDS programs^[6]^. The private sector’s involvement in supply chain management is also critical for ensuring the availability and distribution of HIV/AIDS medications and supplies. Companies with expertise in logistics and distribution can help streamline the supply chain, reduce costs, and improve the efficiency of delivering HIV treatments to remote and underserved areas. For example, UPS, a global logistics company, has partnered with organizations like the Global Fund to improve the delivery of HIV/AIDS medications in Africa. By optimizing supply chains and leveraging their logistics capabilities, private companies can ensure that life-saving treatments reach those who need them most^[7]^. While the private sector’s contributions to the fight against HIV/AIDS are substantial, there are also challenges and limitations. One significant challenge is ensuring equitable access to HIV treatments and prevention tools. While private companies have made strides in reducing the cost of HIV medications, affordability remains a barrier for many people in low- and middle-income countries. Additionally, intellectual property rights and patent protections can limit the availability of generic versions of newer HIV drugs, posing challenges for scaling up access. Addressing these issues requires ongoing collaboration between governments, the private sector, and international organizations to balance innovation with affordability and access^[8]^. Another challenge is the need for greater coordination and alignment between private sector initiatives and national HIV/AIDS strategies. While private companies can bring valuable resources and expertise, their efforts must be integrated with broader public health goals and policies. Ensuring private sector contributions complement and support national strategies requires effective stakeholder coordination and communication. This includes aligning private sector initiatives with the priorities identified in national strategic plans and leveraging private sector resources to fill gaps and address critical needs^[9]^. Despite these challenges, the potential for private sector contributions to the fight against HIV/AIDS remains significant. Continued investment in R&D, particularly for innovative prevention and treatment methods, is crucial for advancing the global response. PPPs that leverage the strengths of both sectors can drive progress and improve health outcomes. Additionally, the private sector’s ability to innovate and scale solutions quickly can be harnessed to address emerging challenges, such as reaching key populations and addressing social determinants of health. Looking forward, there are several opportunities for enhancing private sector contributions to the HIV/AIDS response. One area of potential growth is developing and implementing digital health technologies. The private sector, particularly technology companies, can be pivotal in developing digital tools for HIV prevention, diagnosis, and treatment adherence. For example, mobile health applications and telemedicine platforms can improve access to HIV services, especially in remote areas. Digital technologies can also enhance data collection and monitoring, enabling more effective targeting of interventions and improving the overall efficiency of HIV programs^[10]^. Another opportunity is expanding private sector engagement in financing mechanisms for HIV/AIDS programs. Innovative financing models, such as social impact bonds and blended finance, can mobilize additional resources for HIV/AIDS initiatives. These models leverage private capital to achieve social outcomes, with returns linked to achieving specific targets. By engaging private investors and financial institutions, such mechanisms can provide sustainable funding for HIV programs and drive accountability and performance. Exploring and scaling these financing models can help bridge funding gaps and ensure the long-term sustainability of HIV/AIDS responses^[11]^. Strengthening corporate engagement in advocacy and policy dialogue is essential for maximizing private sector contributions. Companies can use their influence to advocate for policies that support the HIV response, such as increasing funding for HIV programs, protecting the rights of people living with HIV, and addressing social determinants of health. By participating in policy discussions and leveraging their networks, private companies can contribute to creating an enabling environment for effective HIV/AIDS interventions. Fostering multi-sectoral partnerships that include government, civil society, and the private sector can enhance collective action and drive progress towards global HIV/AIDS targets^[12]^.

Community-based organizations (CBOs) and NGOs play a pivotal role in the implementation of HIV/AIDS programs. These organizations are often at the forefront of outreach efforts, providing education, testing, and linkage to care. For instance, the Thai Red Cross AIDS Research Centre has successfully implemented a community-based HIV testing program known as the “Key Population-Led Health Services” (KPLHS), which has significantly increased testing rates among MSM and transgender women. By employing members of the communities they serve, such programs build trust and improve service uptake^[1]^. A critical component of community-based approaches is peer education. Peer educators, who are often members of the target population, use their shared experiences and understanding to educate others about HIV prevention and treatment. This method has proven particularly effective in reaching marginalized groups, such as sex workers, PWID, and MSM. For example, the Sonagachi Project in Kolkata, India, utilizes peer educators to reach sex workers with HIV prevention messages, condoms, and referrals to health services. This project has been credited with reducing HIV transmission rates and empowering sex workers through education and community mobilization^[2]^. Community-based HIV testing and counseling (HTC) is another cornerstone of these approaches. These services are often more accessible and acceptable to people who may not use conventional healthcare facilities due to stigma or logistical barriers. Mobile testing units, community health fairs, and home-based testing are examples of how communities can be engaged in HTC. A study conducted in South Africa found that community-based HTC significantly increased the number of individuals who knew their HIV status and linked it to care compared to facility-based testing alone. This approach helps to identify HIV-positive individuals early and reduces the spread of the virus through timely treatment initiation^[3]^. Treatment adherence support is crucial for ART success. Community-based adherence support groups provide a platform for people living with HIV (PLHIV) to share their experiences, receive encouragement, and solve problems related to treatment adherence. These groups often operate in resource-limited settings where healthcare systems are strained. In Mozambique, for instance, community adherence groups (CAGs) have been implemented to support ART adherence. Members of these groups take turns collecting medication from health facilities, ensuring all members receive their treatment on time. This collective approach has been shown to improve adherence rates and reduce the burden on healthcare facilities^[4]^. Community-based approaches also play a significant role in addressing the social determinants of health that contribute to HIV vulnerability. These determinants include poverty, lack of education, gender inequality, and stigma. Programs incorporating economic empowerment, education, and advocacy can address these underlying issues and create a more supportive environment for HIV prevention and care. The “Stepping Stones” program in sub-Saharan Africa is an example of an intervention that combines HIV education with gender equality and communication skills training. This program has been shown to reduce risky sexual behavior and increase mutual respect and communication within relationships, ultimately contributing to lower HIV transmission rates^[5]^. Stigma and discrimination remain significant barriers to effective HIV prevention and treatment. Community-based initiatives are well-positioned to combat stigma through education, advocacy, and fostering supportive environments. The “Positive Living” program in Uganda, which involves PLHIV sharing their stories and experiences publicly, has been effective in reducing stigma and encouraging more people to get tested and seek treatment. By humanizing the face of HIV, such programs help to break down prejudices and misconceptions about the disease^[6]^. Youth engagement is a critical aspect of community-based approaches, given the high rates of new HIV infections among young people. Peer-led initiatives focusing on youth-friendly services and education can effectively reach this demographic. In Zimbabwe, the “Sista2Sista” program engages young women through mentorship and peer education, addressing issues such as sexual and reproductive health, HIV prevention, and gender-based violence. Programs like these provide vital health information and empower young people to make informed decisions about their health and well-being^[7]^. The involvement of key populations – such as MSM, sex workers, PWID, and transgender individuals – in the planning and implementation of HIV programs ensures that these initiatives are relevant and responsive to their needs. The “MSMGF (Global Forum on MSM & HIV)” works globally to ensure that MSM are meaningfully involved in the response to HIV. By advocating for the rights and health needs of MSM, the organization helps to tailor HIV services to be more accessible and effective for this population. Ensuring that key populations are involved at all program design and implementation stages is crucial for successful community-based approaches^[8]^. Community-based monitoring and evaluation (M&E) are essential for assessing the effectiveness of HIV programs and ensuring accountability. By involving community members in M&E processes, programs can gather more accurate data and insights into the local context. For instance, the “Community-Led Monitoring” initiative by the International Treatment Preparedness Coalition (ITPC) empowers PLHIV to monitor the quality and accessibility of HIV services in their communities. This approach improves program effectiveness and empowers communities to advocate for better services and hold providers accountable^[9]^. Despite the many successes of community-based approaches, there are also significant challenges. Funding is a major issue, as many community-based organizations rely on donor funding, which can be unpredictable and subject to shift priorities. Ensuring sustainable financing for community-based HIV programs is essential for their long-term success. Additionally, capacity building is needed to strengthen the skills and capabilities of community organizations to manage and deliver HIV services effectively. This includes training in program management, monitoring and evaluation, and advocacy^[10]^. Another challenge is the integration of community-based approaches with national health systems. While community programs can be highly effective, they need to be part of a broader, coordinated response to HIV/AIDS. This requires strong partnerships between community organizations, governments, and international agencies. For example, in Kenya, integrating community health workers into the national health system has improved the reach and quality of HIV services. These workers bridge the community and healthcare facilities, ensuring that services are accessible and culturally appropriate^[11]^. Addressing legal and policy barriers is also crucial for the success of community-based approaches. In many countries, laws and policies that criminalize key populations or restrict the activities of NGOs can hinder the effectiveness of HIV programs. Advocacy efforts are needed to reform these laws and create an enabling environment for community-based initiatives. For example, legal reforms in Nepal that decriminalized homosexuality and recognized the rights of transgender individuals have facilitated the implementation of more inclusive and effective HIV programs^[12]^. Looking forward, there are several opportunities to enhance community-based approaches to HIV/AIDS. Digital technologies, such as mobile health applications and social media, can expand the reach of HIV prevention and treatment messages. These tools can provide discreet and accessible information, support, and linkage to services, particularly for young people and key populations. In Nigeria, the “MyQuestion” service uses SMS to provide anonymous HIV counseling and information, reaching thousands of young people who might otherwise lack access to these services^[13]^. Strengthening community engagement in research is another important area. Community-based participatory research (CBPR) involves communities in the design and conduct of research, ensuring that studies are relevant and beneficial to those affected by HIV. This approach can lead to more effective interventions and policies that reflect the lived experiences of communities. For example, the “PARTNER” study, which examined the risk of HIV transmission among serodiscordant couples, involved significant input from community members in its design and implementation, resulting in findings that were highly relevant to the target population^[14]^.

### Strategies for sustainability and cross-cutting issues

The fight against HIV/AIDS has made significant strides in recent decades, primarily due to global investments in prevention, treatment, and care. However, the sustainability of these efforts remains a critical challenge. As the international community continues to confront HIV/AIDS, new strategies must be devised to secure long-term funding and address the pressing cross-cutting issues that influence the effectiveness of interventions^[10-12]^. Recommendations for future funding strategies must prioritize innovative and adaptable models that can withstand economic fluctuations, political changes, and evolving social contexts. A key strategy is the diversification of funding sources. Historically, funding for HIV/AIDS has largely relied on government allocations, international organizations, and private philanthropic contributions. While these sources have provided substantial support, they are often vulnerable to shifts in global priorities, economic crises, and political instability^[13-15]^. Future funding models should incorporate a broader range of stakeholders, including the private sector, NGOs, and local community organizations, to mitigate these risks. By leveraging the strengths of different sectors, such as the flexibility of the private sector and the grassroots reach of NGOs, funding can become more resilient to external shocks. PPPs offer one potential solution: fostering collaboration between government agencies and private companies to pool resources, share risks, and maximize impact. Additionally, integrating local and national funding mechanisms can help ensure that HIV/AIDS programs are sustainable and contextually relevant, allowing for greater ownership and long-term commitment from affected communities^[16-18]^.

Another recommendation is increased investment in preventive measures alongside treatment and care services. While significant progress has been made in the availability of ART, prevention programs often must be funded and prioritized. Prevention is crucial to reducing new HIV infections, and thus, future funding strategies should emphasize a balanced approach that addresses both treatment and prevention^[19-21]^. This includes funding for education campaigns, condom distribution, needle exchange programs, and HIV testing services. Furthermore, integrating HIV prevention into broader public health initiatives, such as maternal and child health or sexual and reproductive health services, could help reduce fragmentation and improve cost-effectiveness^[22,23]^.

Challenges in implementing sustainable funding models are numerous and multifaceted. One significant challenge is the need for more stable and predictable financial commitments. Many HIV/AIDS programs are dependent on donor funding, which is often inconsistent or insufficient for long-term planning. The withdrawal of donor funding or changes in priorities can lead to service disruptions and hinder the progress of ongoing initiatives. For example, the Global Fund to Fight AIDS, Tuberculosis, and Malaria, while successful in mobilizing resources, has faced difficulties in maintaining funding levels in the face of competing global health challenges. To address this, strategies should focus on building more predictable funding mechanisms. This could include the establishment of multi-year funding agreements, endowment funds, or national-level HIV/AIDS trust funds that can provide steady financial support over time^[24-26]^.

Additionally, many countries face challenges in aligning HIV/AIDS funding with national health priorities. This misalignment can lead to inefficiencies and a lack of coordination between international and local efforts. In some cases, there is insufficient political will to allocate adequate funding for HIV/AIDS programs, particularly in countries where the epidemic is not perceived as a national priority. To overcome this, advocates must continue to push for HIV/AIDS to be seen as a critical issue within national health agendas. Strengthening advocacy efforts and ensuring that HIV/AIDS is integrated into broader health and development goals can increase political commitment and secure the necessary resources for sustainable programming^[27-29]^.

Monitoring and evaluation (M&E) are fundamental to ensuring the effectiveness of HIV/AIDS programs and the sustainability of funding strategies. With rigorous M&E systems, it is easier to assess the impact of interventions, identify gaps, and make informed decisions about resource allocation. Developing robust M&E frameworks is critical for tracking progress towards HIV/AIDS targets, such as those outlined in the United Nations SDGs. These frameworks should include process indicators (e.g., program implementation and service delivery) and outcome indicators (e.g., changes in HIV incidence, mortality, and quality of life). Furthermore, community-level involvement in M&E can help ensure that programs are tailored to local needs and are responsive to changing contexts. By incorporating feedback from the affected populations, HIV/AIDS interventions can be continuously improved, and resources can be better targeted to where they are needed most^[29-31]^.

The role of research and development (R&D) in HIV/AIDS funding is another critical area that cannot be overlooked. Research is vital in developing new prevention technologies, treatment regimens, and diagnostic tools. The continued investment in R&D is essential for addressing emerging challenges, such as drug resistance, co-infections, and the need for more affordable treatment options. Additionally, research into the social, economic, and behavioral factors that contribute to the spread of HIV can help inform more effective and culturally appropriate interventions. To sustain R&D funding, creating partnerships between public and private sector entities and international research organizations is crucial. Such collaborations can pool resources, foster innovation, and ensure that research findings are translated into practical solutions^[32-34]^.

Advocacy and public awareness are also critical components of successful HIV/AIDS funding strategies. Advocacy plays a central role in raising the visibility of the epidemic, mobilizing financial and political support, and ensuring that the needs of affected communities are prioritized. Over the years, advocacy efforts have led to significant policy changes, such as the scaling up of ART and the reduction of HIV-related stigma^[35-37]^. However, there is still much work to be done. Advocacy campaigns must continue to emphasize the importance of sustainable funding, the need for more equitable access to HIV/AIDS services, and the elimination of discriminatory practices that prevent individuals from seeking care. Public awareness campaigns are equally crucial for changing social norms, reducing stigma, and encouraging people to seek testing and treatment. In this regard, the media and social platforms can be powerful tools for spreading information and mobilizing action^[38]^.

Ethical considerations in HIV/AIDS funding are paramount to ensuring that interventions are not only effective but also equitable and just. The allocation of resources must prioritize the most vulnerable populations, including women, children, and key affected groups such as MSM, sex workers, and PWID^[39,40]^. Ethical issues also arise in the context of access to treatment, with concerns about affordability, quality, and the right to health. In addition, there are important ethical questions surrounding the use of data in HIV/AIDS programs. The protection of individual privacy and the informed consent process must be central to any data collection efforts, particularly as HIV-related data can be sensitive and subject to misuse. Donors, governments, and implementing organizations must work together to ensure that their funding strategies align with human rights principles and prioritize the well-being of affected individuals and communities^[42-44]^.

### Unique contribution and gaps in existing literature

The unique contribution of this work lies in its comprehensive examination of innovative funding mechanisms for HIV/AIDS, with a particular focus on their sustainability, scalability, and equity. While existing literature often addresses specific funding models in isolation, this review provides a comparative analysis of PPPs, social impact bonds (SIBs), and blended finance mechanisms, critically assessing their successes, challenges, and relevance to low-resource settings. This integrated approach fills a significant gap in the literature, as few studies offer a detailed synthesis of these diverse funding mechanisms in the context of HIV/AIDS. The review also introduces a novel framework for evaluating these funding mechanisms, incorporating sustainability, scalability, and equity dimensions. This framework allows for a more holistic assessment of each model’s effectiveness in addressing the long-term funding needs of HIV/AIDS programs, particularly in resource-constrained environments. Considering these key factors, the review provides valuable insights into how different mechanisms can be leveraged to ensure effective and equitable funding.

Additionally, the analysis highlights the potential of newer financing mechanisms, such as SIBs and blended finance, to attract private sector investment and improve the efficiency and impact of HIV/AIDS programs. A notable gap in the literature that this review addresses is the need for more focus on sustainability strategies for funding in low-resource settings. While many studies highlight the challenges of donor-dependent funding models, only some propose viable alternatives that reduce reliance on external aid. This review examines how domestic resource mobilization, PPPs, and blended finance can help countries build more sustainable and resilient funding systems. The review must also fill a gap in the fragmented approach to studying innovative funding models. Previous research often examines these mechanisms theoretically without addressing the real-world challenges that arise during implementation. For instance, while PPPs are widely recognized as a promising solution for mobilizing resources, their practical challenges, such as governance issues and accountability risks, should be explored in more detail.

Similarly, although SIBs have garnered attention as a way to incentivize outcomes-based funding, their feasibility and scalability in the HIV/AIDS context are underexplored. This review bridges these gaps by providing a comparative analysis of the structures, successes, and limitations of PPPs and SIBs, exploring their potential to complement each other in HIV/AIDS financing. Another significant gap identified is the lack of comprehensive literature on how blended finance can be adapted for HIV/AIDS funding. Blended finance models, which combine public and private funding, have the potential to unlock new sources of capital and foster innovation in the HIV/AIDS sector. However, existing studies rarely assess how these models can be tailored to the specific needs of the epidemic. This review contributes to the literature by providing an in-depth analysis of blended finance in the HIV/AIDS context, emphasizing the importance of aligning public and private sector interests to achieve equitable and sustainable outcomes.

Furthermore, the review introduces innovative perspectives on aligning funding mechanisms with regional priorities. While many funding models are discussed globally, this review emphasizes the need to tailor financing mechanisms to the unique health challenges faced by different regions. For example, the review explores how SIBs could be customized to address specific health needs in low-income countries, considering local context and priorities.

### Final remarks

The journey towards sustainable HIV/AIDS funding is both a challenge and a beacon of hope. As we reflect on the myriad strategies discussed – from innovative funding models to community-based approaches – it becomes evident that no single approach holds the key. Instead, the synergy of these efforts, driven by ethical considerations, will pave the way forward. The commitment of international organizations, governments, NGOs, and the private sector is crucial in sustaining progress. Advocacy and public awareness remain formidable allies, breaking down stigma and mobilizing support. Technological innovations continue to reshape the landscape, making healthcare access more equitable and information dissemination more effective. Yet, amidst these triumphs, challenges persist. Political will and global solidarity must be fortified to ensure funding commitments endure beyond short-term cycles. The ethical imperative to prioritize marginalized communities and uphold human rights must remain steadfast.

### Call to action

Looking ahead, as we continue to innovate, collaborate, and advocate, we are reminded that the fight against HIV/AIDS is not just about medical breakthroughs or financial pledges – it is about empathy, resilience, and a shared vision of a world where health equity prevails. Together, we can shape sustainable paths for HIV/AIDS funding, ensuring that no one is left behind in our pursuit of a healthier, more just future.

## Data Availability

This published article and its supplementary information files include all data generated or analyzed during this study.
